# Structural and functional analysis of the *Bacillus cereus* GerI inosine-responsive spore germinant receptor

**DOI:** 10.1128/mbio.00108-26

**Published:** 2026-03-27

**Authors:** Yunfeng Li, Giannina Ow-Young-Villarreal, Yulia Pustovalova, David M. D. Bailey, Joshua Yarrow, George Korza, Faith Ye, Heidi Erlandsen, Peter Setlow, Graham Christie, Bing Hao

**Affiliations:** 1 Department of Molecular Biology and Biophysics, UConn Health 705913, Farmington, Connecticut, USA; 2 Department of Chemical Engineering and Biotechnology, University of Cambridge 2152, Cambridge, United Kingdom; 3 Department of Zoology, University of Cambridge, Cambridge, United Kingdom; 4 Department of Pharmaceutical Science, University of Connecticut 7712, Storrs, Connecticut, USA; Prairie View A&M University, Prairie View, Texas, USA

**Keywords:** spore germination, germinant receptor, *Bacillus cereus*, structural biology, *Bacillus*

## Abstract

Bacterial endospores are metabolically dormant yet can rapidly return to vegetative growth upon exposure to nutrients through the process of germination. Spore germination is triggered by specific chemical nutrients binding to cognate germinant receptors (GRs) in spores’ inner membrane. These GRs function as ligand-gated ion channels and are composed of clusters of at least three subunits. Given their central role in germinant recognition and discrimination, elucidating 3D structures of GR subunits is a key part of efforts to understand the mechanism(s) of spore germination. Here, we present the crystal structure of the N-terminal domain of the *Bacillus cereus* GerIA protein (GerIA^NTD^), a component of the inosine-responsive GerI GR. GerIA^NTD^ adopts a conformation homologous to substrate-binding proteins in bacterial ABC transporters. NMR chemical shift perturbation and site-directed mutagenesis identified GerIA^NTD^ residues potentially involved in inosine binding or critical for germinosome assembly in *B. cereus* spores, modification of which abrogated inosine-induced germination. Molecular modeling and mutagenesis additionally identified residues in the GerIB subunit forming germinant and cation-binding sites. GerQ, the second GR that contributes to inosine germination in *B. cereus* spores, was capable of complementing hypomorphic *gerI* alleles in several instances, demonstrating cooperative restoration of function despite being incapable of initiating germination to inosine in *gerI* null spores. Collectively, our results provide new insights into GR subunit function and the molecular basis of the *B. cereus* germinative response to inosine.

**IMPORTANCE:**

Many bacteria in the order *Bacillota* form spores that survive antibacterial treatments, including antibiotics. However, once these spores germinate and return to growth, they become vulnerable to antibiotics and other treatments. Notably, growing cells of some of these species cause food spoilage or serious diseases. Thus, there is much interest in spore germination, as stimulating this process would allow for easy spore eradication. This study has investigated precisely how spores’ germinant receptors (GRs) recognize and respond to triggers of spore germination, such as inosine and L-alanine. Using a combination of structural biology, computational modeling, and functional assays with targeted GR mutations, our work uncovered new insights into GR function and the initiation of germination. These findings not only advance our understanding of a critical biological process but also provide new directions for spore control strategies.

## INTRODUCTION

Spores of *Bacillus* species, while dormant and able to survive in this state for years due to their resistance, constantly sense the environment (1, 2). Thus, when conditions suggest vegetative growth is possible, spores respond to the presence of specific low molecular weight molecules and rapidly return to life via the process of germination (3, 4). The molecules that trigger this process are termed germinants, generally amino acids, monosaccharides, and/or purine nucleosides, and are sensed by germinant receptors (GRs) in spores’ inner membrane (IM). GRs commonly contain three subunits, A, B, and C, all essential for GR function and in one case, two GRs cooperate to respond to a germinant mixture (5–7). While all GR subunits are associated with spores’ IM, their specific IM location differs between subunits. The C subunit is a lipid-anchored, peripheral IM protein, the B subunit is largely an integral IM protein, with the A subunit having both hydrophilic and integral IM components (8, 9). Some GRs contain a small D-subunit, which can modulate GR activity (10). Importantly, GRs are also found together in one or two large, organized IM complexes termed germinosomes, which are scaffolded by the IM lipoprotein GerD (11–13).

**Fig 1:**
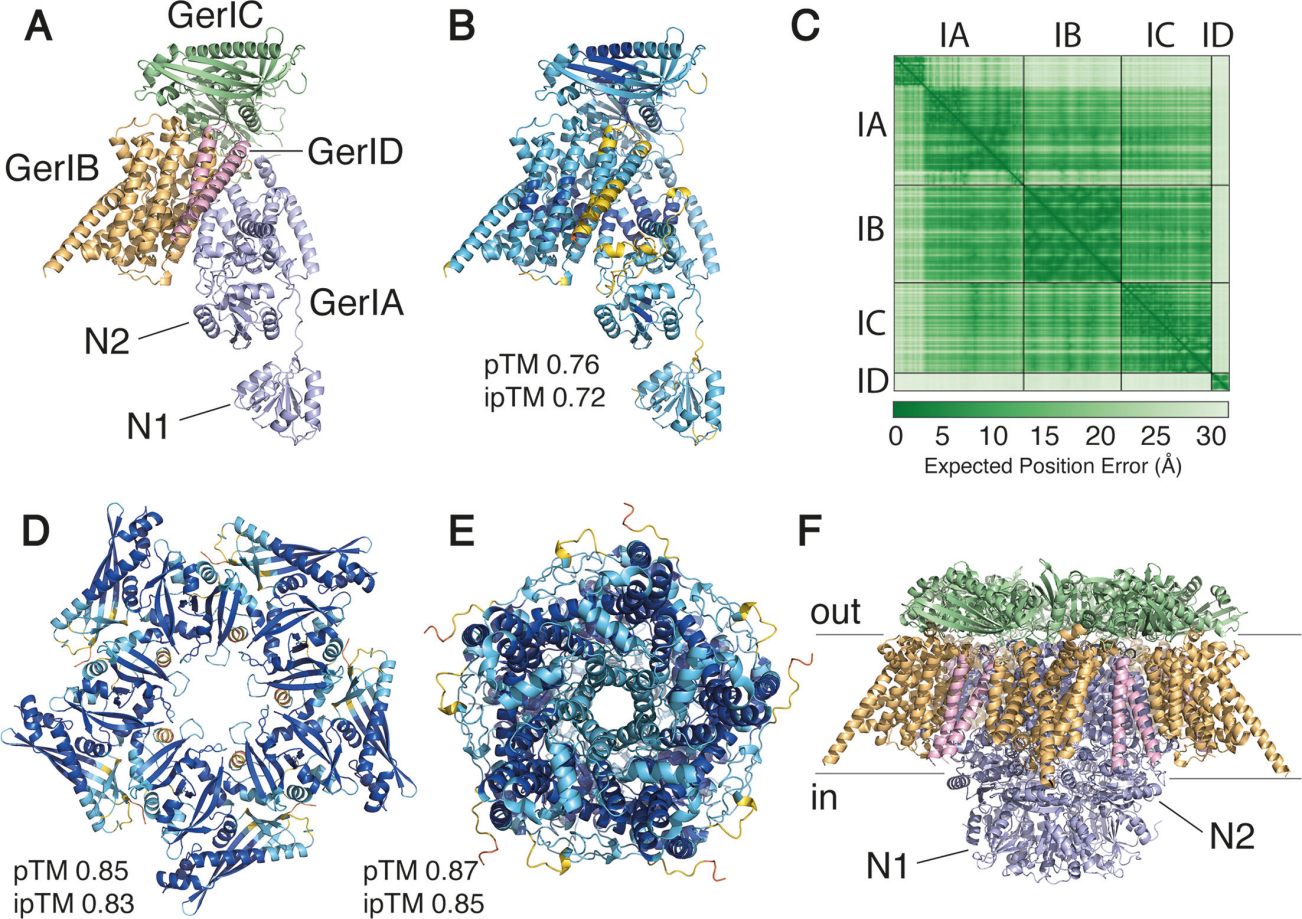
Structural model of the *Bacillus cereus* ATCC 14579 GerI GR. (**A**) AlphaFold 3 model of the GerI protomer, with GerIA in gray, GerIB in orange, GerIC in green, and GerID in pink, respectively. Residues 1–244 of GerIA, which are predicted to be disordered, are not shown. GerIA^NTD^ subdomains N1 and N2 (residues 245–500) are labeled. (**B**) AlphaFold predicted local distance difference tests (pLDDT) per position mapped onto the GerI tetrameric protomer. Mapped pLDDT values range from blue, which are associated with high confidence predictions, progressing through yellow and red for less confident predictions. Template modeling (pTM) and interface predicted template modeling (ipTM) scores are shown. (**C**) Predicted aligned error (Å) for all residues against all residues for the top-ranked protomer model, where low error (green) corresponds to well-defined relative domain positions. (**D**) Top-ranked AlphaFold 3 model of the GerIC pentamer and (**E**) GerIA pentamer, with mapped pLDDT values and pTM and ipTM scores for each structure. (**F**) GerI GR pentamer of the GerIA-IB-IC-ID tetramers model viewed in the plane of the membrane, colored according to panel **A**.

Recent work suggests that GRs function as ligand-gated ion channels (14). Structural models generated by AlphaFold predict that GRs assemble as multimers consisting of five or six GR protomers, with C subunits forming a ring on the outer IM surface while the A subunits’ N-terminal hydrophilic domain (A-NTD) and small C-terminal tail are on the IM’s inner surface (14, 15) (Fig. 1). As expected, hydrophobic regions of A and B subunits are predicted to span the IM. In support of these models, data from accessibility and topology studies of GR subunits in *Bacillus anthracis* (16) and *Bacillus subtilis* (17) showed that A-NTDs are in the spore core. Interestingly, one of these studies found that the C subunit of at least one *B. subtilis* GR is largely inaccessible to an exogenous chemical agent in decoated dormant but not germinated spores (17).

Given the central role of the GRs in germinant recognition and discrimination, elucidating experimental three-dimensional structures of GR subunits has become key in understanding GR function. Progress in this regard to date has been limited to crystal structures of the C subunit of the *B. subtilis* GerB GR (GerBC) (18) and to the A-NTD of a hypothetical *Bacillus megaterium* GerK_3_ GR (GerK_3_A^NTD^) (19). While the former adopted a previously uncharacterized type of protein fold consisting of three distinct mixed α/β domains, GerK_3_A^NTD^ was shown to exhibit strong structural similarity to bacterial periplasmic binding proteins (PeBPs) that are receptors for membrane-associated small-molecule transporters and signal transducers, albeit these proteins differ in the topological arrangement of secondary structure elements in their subdomains (19).

With this relative paucity of experimental structural information in mind, the current study examines the analogous A-NTD of the *Bacillus cereus* GerI GR, having previously established that it can be expressed in a soluble and stable form and therefore might be amenable to X-ray crystallography and NMR spectroscopy studies (19, 20). The GerI GR is of particular interest since it has a prominent role in the *B. cereus* spore germination response to inosine, a property that is shared with spores of other members of the *B. cereus sensu lato* family (21). The orthologous GerH GR in *Bacillus anthracis*, for example, shares >95% sequence identity across the core regions of the respective GR subunits with GerI. However, while *B. cereus* spores can germinate in response to inosine as a sole germinant, *B. anthracis* spores require the additional presence of one of several amino acids to act as co-germinants (22, 23). *B. cereus* spores also differ from other members of the wider *B. cereus sensu lato* family in that they have a second inosine-responsive GR, termed GerQ, which appears to act in concert with GerI to trigger efficient inosine-mediated germination (24). GerQ is quite distinct phylogenetically from GerI and GerH, sharing <45% amino acid identity across the core regions of the respective GR subunits. Furthermore, detailed kinetic studies conducted with libraries of purine ribonucleoside analogs revealed quite different patterns of stimulatory and antagonistic germinative responses mediated by GerI and GerQ, with the latter seemingly being cognate for a much wider range of ligands than GerI (25, 26).

Given the biological significance and at times contradictory information in the literature concerning the sensing of inosine as a germinant, the purpose of this study was to integrate existing knowledge with new structural and functional insights, principally to the *B. cereus* GerI GR. Presented data reveal probable binding sites for inosine and co-germinants within the GerI complex, while we additionally identify regions of the GR that are of functional or structural importance and which ultimately influence inosine-triggered spore germination.

## RESULTS

### Crystal structure of the A-NTD of *B. cereus* GerI GR

**Fig 2:**
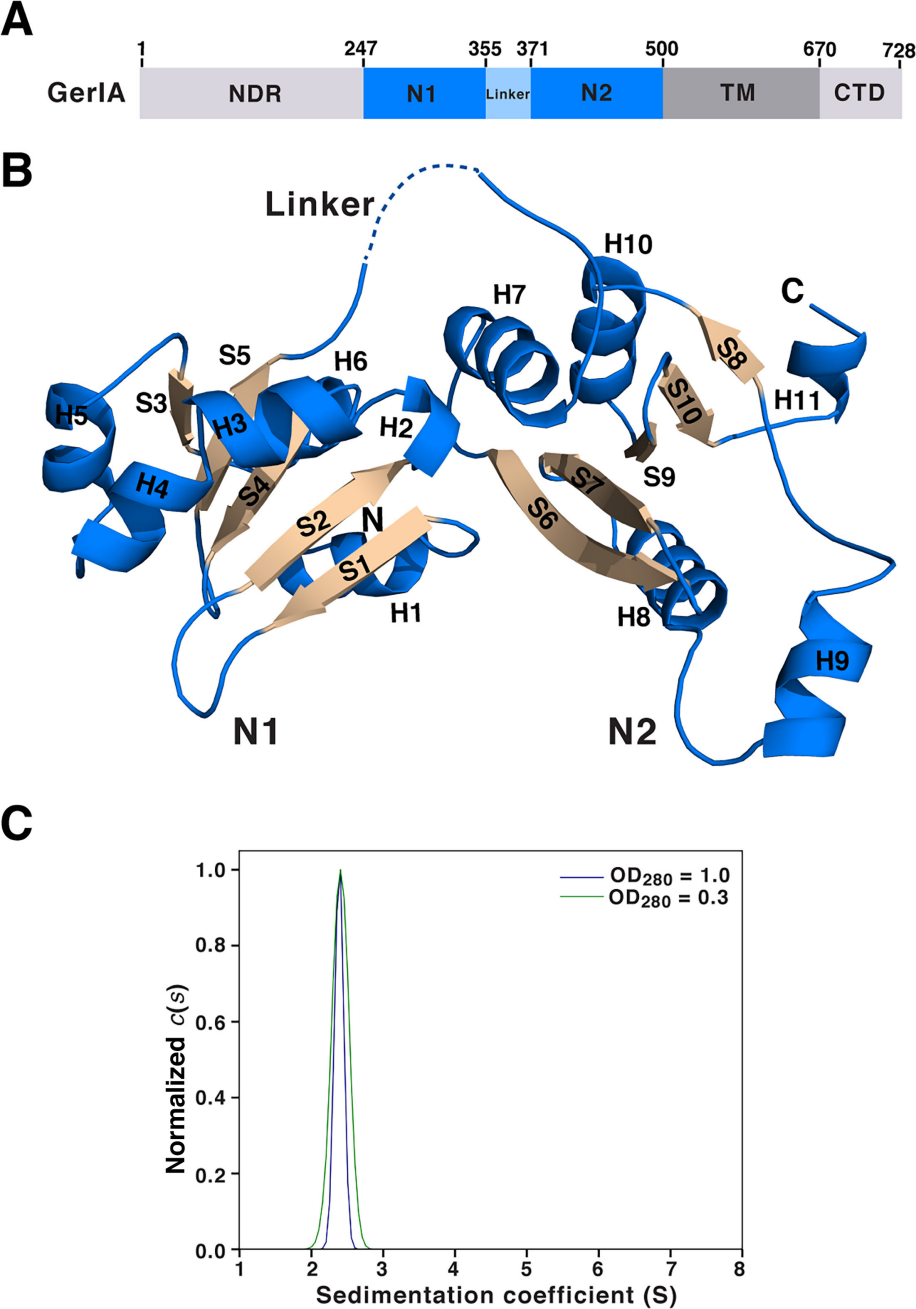
Crystal structure and oligomerization state of GerIA^NTD^. (**A**) Schematic representation of the domain organization of *B. cereus* ATCC 14579 GerIA. GerIA^NTD^ (residues 245–500) used in this study comprises two subdomains (N1 and N2) and a flexible linker. (**B**) Ribbon diagram of GerIA^NTD^, with the secondary structure elements labeled. α helices and β strands are shown in blue and orange, respectively. The disordered linker is marked by a dashed line. (**C**) Sedimentation velocity analysis of GerIA^NTD^ loaded at two protein concentrations as indicated. The sedimentation velocity traces were analyzed using SEDFIT (28) to obtain *c*(*s*) distributions with the peak near s = 2.38 S and were normalized by height.

As alluded to above, in order to explore the structure-function relationship of a functional GR A subunit, we selected the conserved A-NTD of *B. cereus* ATCC 14579 GerI GR (GerIA^NTD^; residues 245–500; 29.4 kDa) given the essential role of GerI in the inosine-mediated germination of *B. cereus* spores (21, 24, 27) (Fig. 2A). Note that the GerIA^NTD^ construct used in our study lacks the ~240 amino acid N-terminal disordered region (NDR) whose function remains unknown (a similar NDR spanning ~260 amino acids is present in *B. anthracis* GerHA). The crystal structure of GerIA^NTD^ was determined using the multiple-wavelength anomalous dispersion (MAD) method, with data collected at the selenium peak and edge wavelengths (Table S1). GerIA^NTD^ adopts a butterfly-shaped architecture comprising two globular subdomains (N1 and N2), connected by a largely disordered linker in the crystal structure (residues 356–372; Fig. 2B). Each subdomain features a central five-stranded antiparallel β sheet flanked by α helices on both sides. As expected, the structure of GerIA^NTD^ closely resembles that of GerK_3_A^NTD^ (4). Despite modest sequence identity (28.4%), the two proteins superimpose with an overall root-mean-square deviation (RMSD) of 2.2 Å across 183 Cα atoms (Fig. S1A). The primary structural differences lie in loop lengths within N1 and positions of helices H9 and H10 in N2. Like GerK_3_A^NTD^, despite low sequence identity (2.4% to OpuAC), GerIA^NTD^ bears structural resemblance to PeBPs (3.2 Å of Cα RMSD), though GerIA^NTD^ adopts a different sequential order of the secondary structure elements in both subdomains (Fig. S1B). Our findings support the notion that despite species-specific differences and germinant preferences, the A-NTDs of GRs share a conserved core architecture.

### Analytical ultracentrifugation analysis of GerIA^NTD^

Both AlphaFold2 multimer (29, 30) and AlphaFold 3 (31) predict with high confidence that the GerIA-GerIB-GerIC trimer assembles into a pentameric or hexameric complex, resembling the archetypal *B. subtilis* GerA GR (14) (Fig. 1). AlphaFold 3 additionally incorporates the small GerID subunit, encoded within the *B. cereus gerI* operon, into this model with high confidence (Fig. 1). Notably, the predicted model shows extensive protomer–protomer interactions involving both GerIA^NTD^ and GerIC. However, previous sedimentation velocity and equilibrium ultracentrifugation experiments have demonstrated that *B. subtilis* GerBC is monomeric in solution (and as an interlocked dimer in the crystal structure) (18). Given that the GerIA^NTD^ crystals contain three protein molecules per asymmetric unit, we investigated its oligomerization state in solution. Sedimentation velocity experiments revealed that GerIA^NTD^ is predominantly monomeric in solution, with the velocity *g*(s*) and *c*(*s*) analyses showing a major species at 2.38 S, consistent with the monomer mass of 31.4 kDa (Fig. 2C). Taken together, these results suggest that, like GerBC, GerIA^NTD^ exists exclusively as a monomer in solution. As such, if we assume that the AlphaFold-predicted oligomeric assembly is broadly correct, then such interactions are presumably stabilized by other GR components, most likely the membrane-spanning region of GerIA.

### NMR study of the A-NTD of GerIA and its interaction with inosine

**Fig 3:**
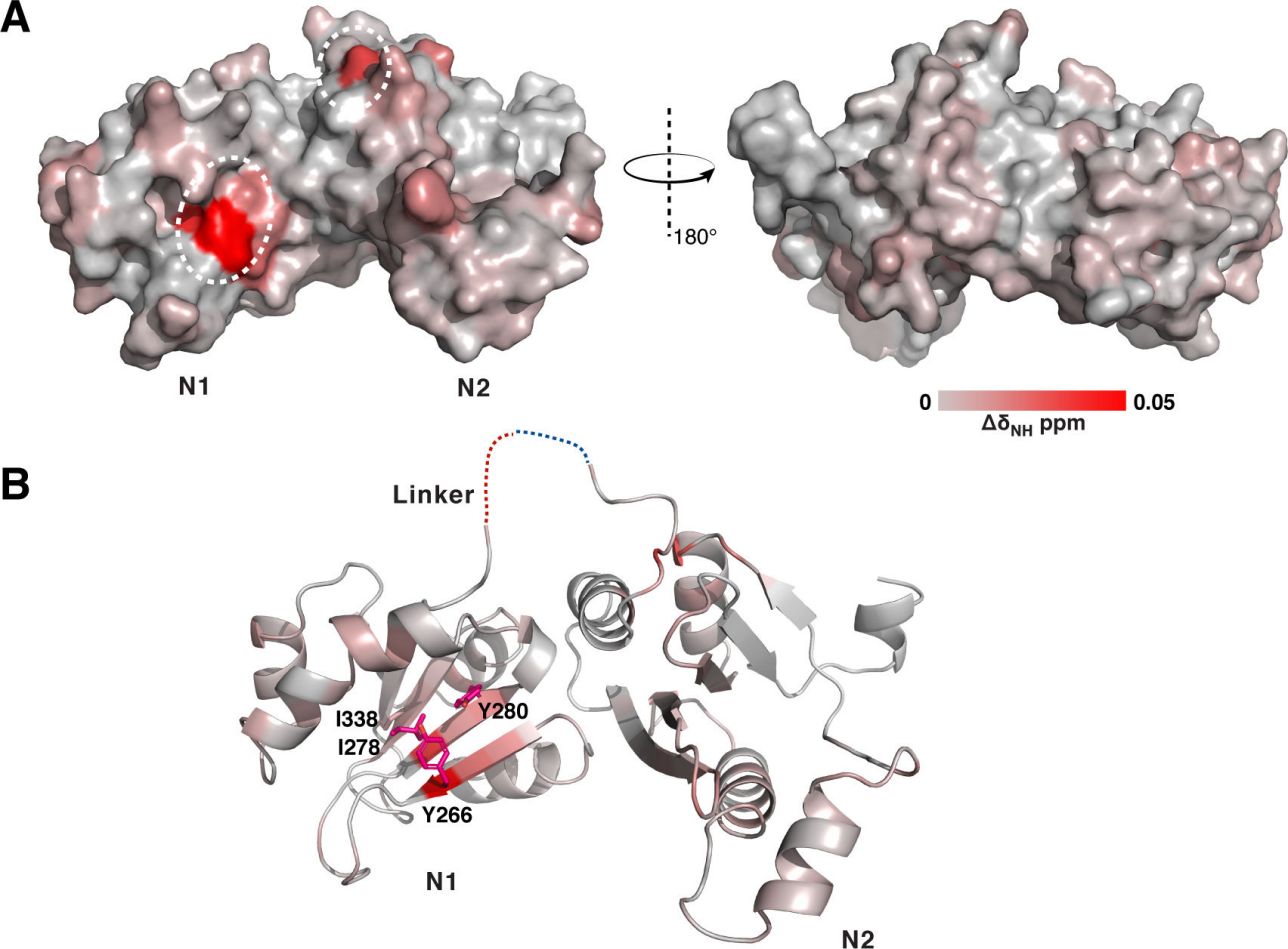
CSP analyses of GerIA^NTD^ in the presence of inosine. (**A**) Surface representation of GerIA^NTD^ colored according to CSPs induced by inosine titration, from light gray (no observed CSP) to red (maximum CSP). The orientation of GerIA^NTD^ shown on the left matches that in Fig. 2B. (**B**) Residues that are selected for site-directed mutagenesis (SDM) studies are shown as licorice sticks. Dashed line indicates the disordered linker.

With the structure of GerIA^NTD^ determined, we sought to identify potential interaction sites with the germinant inosine. As attempts to crystallize the GerIA^NTD^-inosine complex were unsuccessful, we used NMR spectroscopy for further studies. Our previous work showed that GerIA^NTD^ yields a well-dispersed ^1^H–^15^N heteronuclear single quantum coherence (HSQC) spectrum with selective peak shifts upon addition of inosine (19). Because accurate mapping of protein–ligand interactions by NMR requires near-complete backbone resonance assignments, we used a slightly different construct, GerIA^NTD2^ (residues 238–484), which lacks the C-terminal 12 amino acids corresponding to the S10 strand and the H11 helix. GerIA^NTD2^ produced higher quality NMR spectra, making it better suited for backbone chemical shift assignment (20). Chemical shift perturbations (CSPs) caused by inosine titration were measured as per-residue differences in peak positions between the free and inosine-bound forms of GerIA^NTD2^ in HSQC spectra (Fig. S2). Of 162 assigned residues, 21 showed CSPs exceeding two standard deviations (2σ) above the mean (Table S2). Mapping those residues onto the GerIA^NTD^ structure revealed three spatially distinct surface regions affected by inosine binding (Fig. 3A and B): (i) a site around the β sheet in N1, (ii) a surface cavity in N2, and (iii) the linker between N1 and N2. Notably, these sites differ from known ligand-binding regions in PeBPs, suggesting that if GerIA^NTD^ directly interacts with inosine, it may do so via a distinct mechanism. Nevertheless, it is notable that CSPs were only observed at high millimolar inosine concentrations, far exceeding those necessary to stimulate germination, suggesting that the interaction, if direct, is relatively weak. Interestingly, although we have also obtained crystals of GerIA^NTD2^, these crystals have never diffracted beyond 8 Å, implying that the absence of the C-terminal S10 and H11 may compromise proper folding of the NTD and/or its response to inosine.

### Structure-function analysis of GerIA^NTD^

**Fig 4:**
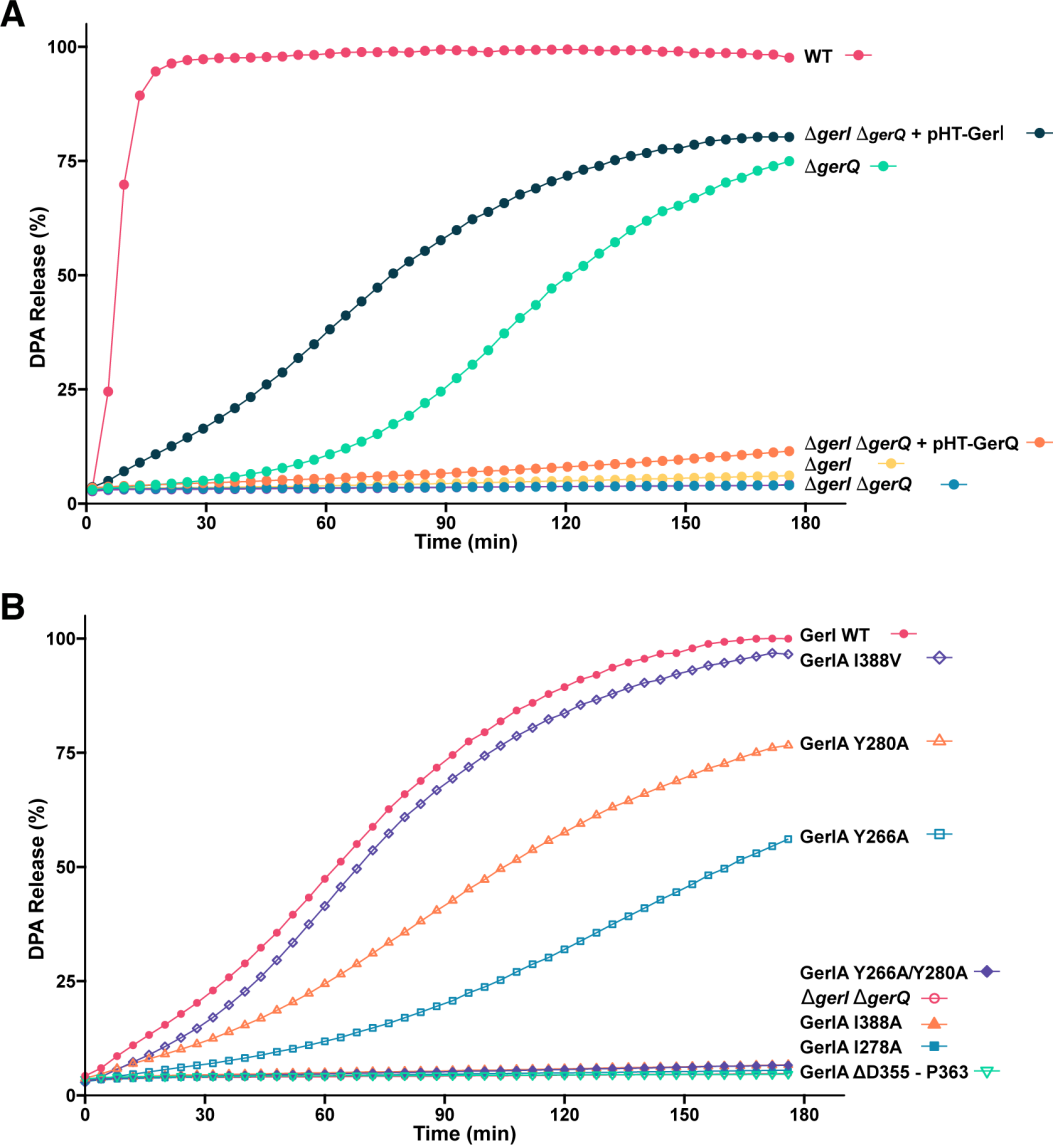
Germination of *B. cereus* GR-null and GerIA mutant spores. (**A**) Germination of *gerI*, *gerQ,* and *gerI gerQ* null mutant spores and their complementation with plasmid-borne wild-type GerI or GerQ. (**B**) Germination of *gerI gerQ* double mutant spores ectopically expressing plasmid-borne GerI with wild-type or variant GerIA-GFP GRs. Spores were heat-shocked (75°C for 30 min) and cooled before resuspending in buffer (50 mM Tris-HCl, 100 mM NaCl, pH 8.0, supplemented with 1 mM inosine and 50 µM TbCl_3_). Germination was monitored using fluorimetric measurements of DPA release as described in the Materials and Methods in the Supplementary material. The presented data are average values from triplicate experiments conducted with the same batch of spores. Similar values were obtained with additional batches of spores. The average standard deviation (SD) from mean values is <10%.

Having mapped HSQC-based CSPs for GerIA^NTD^ titrated with inosine, we next sought to assess the functional significance of the affected residues. This required creating a *B. cereus* strain lacking both *gerI* and *gerQ* operons, which together encode inosine-responsive GRs (21, 24), although only GerI can initiate inosine germination but at a much slower rate compared to the wild-type strain (Fig. 4A). The double mutant strain, which was otherwise isogenic with *B. cereus* ATCC 10876, was then complemented with *gerI* operons bearing appropriate substitutions in the *gerIA* ORF. A low copy number plasmid encoding the *B. cereus* ATCC 14579 *gerI* operon preceded by the native promoter, and with the *gerIA* ORF fused at the 3′ end with *gfp*, served as the basis for site-directed mutagenesis (SDM) analyses. The *B. cereus* ATCC 14579 *gerI* operon was selected for compatibility with the GerIA^NTD^ crystal structure, although the orthologous *B. cereus* ATCC 10876 operon shares a high degree of sequence identity (97%, 91%, and 87% amino acid identity for the respective A, B, and C subunits of GerI). The resultant strains (Table S3) sporulated normally. Furthermore, spores with the GerIA-GFP protein harbored one or two fluorescent germinosomes (Fig. S3) and germinated with kinetics comparable to the analogous non-fluorescent strain (Fig. 4B), indicating that GerIA-GFP and GerI-GFP GR are functional.

GerIA^NTD^ residues subject to SDM were selected primarily based on their CSP rankings and structural context within the crystal structure. Several high-ranking residues, Y266 (rank 1; Table S2), I278 (2), Y280 (7), and I338 (16), are clustered in the β sheet and form a potential ligand binding pocket on the exterior of the N1 subdomain, consistent with the AlphaFold model for GerI (Fig. 1F). Hence, each residue was subject to alanine substitution; I338 was also mutated to valine and a Y266A/Y280A double mutant was prepared. A truncation mutant (D355–P363) that targets residues in the flexible N1–N2 linker, which also showed significant CSPs (Table S2), was also constructed. Spores carrying these GerIA variants were purified and tested for germination with 1 mM inosine using fluorimetric measurements of dipicolinic acid (DPA) release. Alanine substitutions at I278 and I338 caused severe germination defects, while Y266A and Y280A had moderate effects (Fig. 4B). The Y266A/Y280A double mutant and the loop deletion abolished germination. In contrast, the conservative I338V substitution retained function. Importantly, germination phenotypes generally correlated to an extent with germinosome formation, i.e., strong germination defects were associated with diffuse GFP fluorescence, whereas functional variants retained 1–2 germinosome foci per spore (Fig. S3). One notable exception concerns spores with the loop deletion, which retained distinct germinosomes yet failed to germinate, indicating GR assembly commensurate with incorporation into the germinosome but, for reasons that are unknown, loss of function. The Y266A and Y280A spores also formed foci, albeit the fluorescence intensity of the latter was reduced, and were associated with moderate germination defects. Together, these results suggest that some of the residues exhibiting large CSPs in the N1 subdomain of GerIA^NTD^ are structurally important for germinosome assembly and/or participate in inosine-mediated germination. Because receptor levels were not measured in these mutant spores, it remains possible that the absence of germinosomes reflected reduced expression of the GR receptor rather than a direct functional defect.

### Structure-function analysis of GerIB

**Fig 5:**
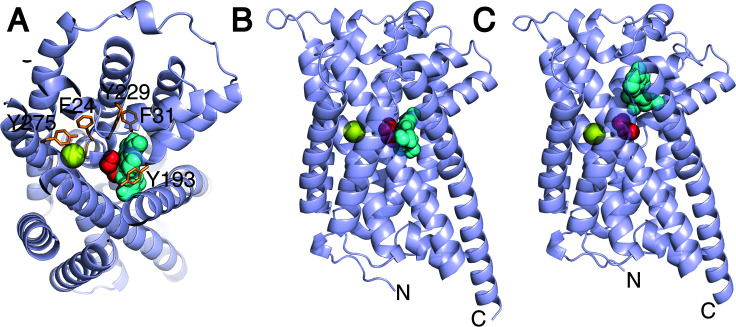
Germinant binding sites in *B. cereus* ATCC 14579 GerI GR. (**A**) AlphaFold model of GerIB (gray) with bound inosine (cyan), L-alanine (red), and Na^+^ (yellow). Germinants were placed by aligning GerIB with crystal structures of ligand-bound Mhp1 (hydantoin and Na^+^) and GkApcT (L-alanine). GerIB residues predicted to reside in germinant- and cation-binding pockets and shown by mutagenesis to be crucial to inosine germination are labeled and shown as sticks. (**B**) AlphaFold model of GerIB with bound germinants viewed in the plane of the membrane, and (**C**) Chai-1 top-ranked model of GerIB with bound germinants.

As presented above, NMR titration and mutagenesis data on GerIA^NTD^ suggest intriguing functional roles, but these are difficult to reconcile with a direct germinant-binding activity for GR A subunits, given their predicted spore core location in the AlphaFold model (Fig. 1). In contrast, the GR B subunits, based on the same model, are ideally positioned to receive germinants that have traversed the outer layers of the spore to the vicinity of the IM. Indeed, structural superposition of AlphaFold models of several GR B subunits, none of which have experimental structures, with crystal structures of APC secondary transporters reveals consistent alignment with an outward-open conformation. This orients the putative ligand-binding sites toward the spore exterior, although we shouldn’t assume this is the conformation adopted in the dormant spore. With this in mind, and in an attempt to identify candidate inosine-binding residues, the GerIB model was superimposed with crystal structures of appropriate ligand-bound APC transporters. Based on structural similarity, the GerIB model most closely resembles Mhp1, the sodium-hydantoin transporter from *Microbacterium liquefaciens* in the nucleobase-cation symport-1 family (32), where it co-transports nucleobases from the growth medium with sodium ions (the electrochemical gradient associated with the latter providing the energy required for substrate transport). Although Mhp1 structures in the holo outward-open conformation have not been determined, GerIB superimposes reasonably well with the apo outward-open structure of the transporter (PDB:2JLN [33]; RMSD of 5.02 Å for 302 Cα pairs spanning TM1-10 of the five-helix inverted repeated core fold) and the ligand-bound occluded structure (PDB:4D1A [34]; RMSD of 6.40 Å for 330 Cα pairs). An inosine molecule was aligned to the hypoxanthine moiety of the hydantoin ligand present in the latter structure, enabling identification of GerIB’s putative inosine binding pocket (Fig. 5A). Moreover, a putative Na^+^ ion was placed in a location analogous to the Na^+^2 site in the LeuT transporter, which is conserved in most APC transporters where crystal structures are available. Similarly, because GerIB also contributes to L-alanine-mediated germination, the structural model was superimposed with the inward-occluded alanine-bound structure of the *Geobacillus kaustophilus* broad-specificity amino acid transporter, GkApcT (PDB:5OQT [35]; RMSD of 4.08 Å for 325 Cα pairs), revealing a putative L-alanine binding site. The GerIB model with bound inosine, L*-*alanine, and a single Na^+^ ion is shown in Fig. 5A. Notably, the putative L*-*alanine binding site is distinct but immediately adjacent to the predicted inosine binding site, raising the possibility of simultaneous binding and germinant cooperativity. Similar docking results were obtained using the Chai-1 structural algorithm (36), which predicted Na^+^ and L-alanine binding sites analogous to those identified by superimposing crystal structures. However, inosine binding was predicted to be more variable, occupying either the L-alanine site (with Na^+^ alone) or a distinct site (with both Na^+^ and L-alanine) (Fig. 5B and C; and see GerQB below). Consideration of the Chai-1 predicted template modeling (pTM) (0.93–0.95) and interface predicted template modeling (ipTM) (0.4–0.6) values suggests high confidence in the overall predicted structures, but less assurance in the relative placement of bound ligands within GerIB.

**Fig 6:**
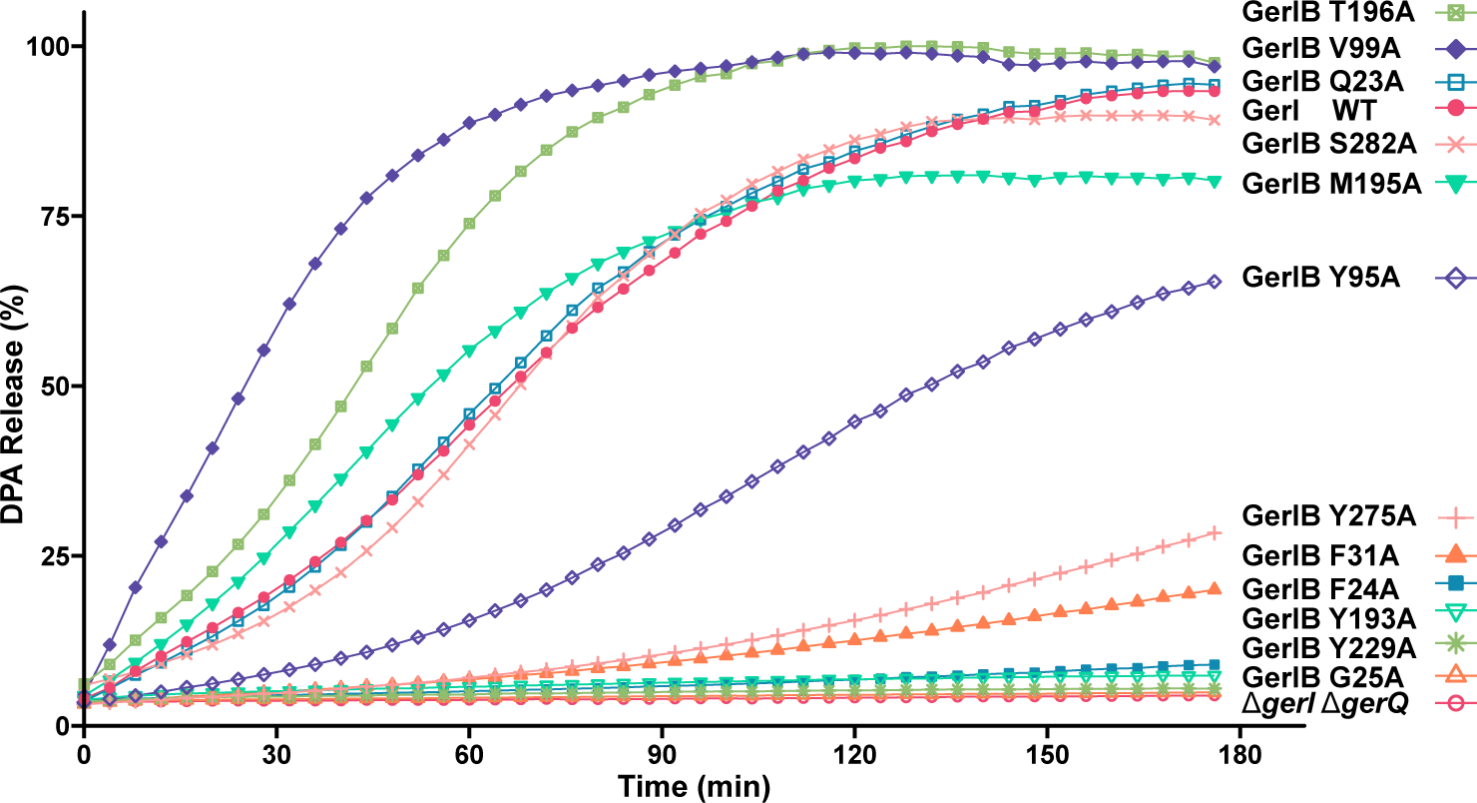
Germination of *B. cereus gerI gerQ* spores ectopically expressing plasmid-borne GerI with wild-type or variant GerIB proteins. Spores were heat-shocked (75°C for 30 min) and cooled before resuspending in buffer (50 mM Tris-HCl, 100 mM NaCl, pH 8.0, supplemented with 1 mM inosine and 50 µM TbCl_3_). Germination was monitored using fluorimetric measurements of DPA release as described in the Materials and Methods in the supplemental material. The presented data are average values from triplicate experiments conducted with the same batch of spores. Similar values were obtained with additional batches of spores. Average SD from mean values is <10%.

Based on these models (Fig. 5A), we identified candidate GerIB residues for mutagenesis studies: (i) F24, Y275, and S282, potentially coordinating Na^+^, and (ii) Q23, F31, Y95, V99, Y193, M195, T196, and Y229, forming the putative inosine-binding pocket. Several residues were chosen for the aforementioned reasons and/or for their predicted proximity to unwound regions of TM1 (Q23, F24, G25) or TM6 (M195, T196), all functionally critical in APC superfamily transporters. Alanine-substituted GerIB variants were introduced to the *B. cereus gerI gerQ* strain expressing GerIA-GFP as described above, and purified spores were assessed for inosine germination. Data from these experiments are generally supportive of the proposed model: first, mutation in TM1 (F24A, G25A) and TM8 (Y275A) severely impaired germination despite normal germinosome formation (Fig. 6; Fig. S4), suggesting that a functionally important cation binding site is located in the vicinity of the unwound region of TM1 and TM8. Second, TM6 substitutions had striking effects: M195A and T196A accelerated germination, while Y193A abolished it, indicating the unwound region of TM6 is clearly of great significance to GerIB function (Fig. 6). Last, other predicted inosine-binding site substitutions yielded a range of phenotypes, from enhanced germination (V99A) to moderate (Y95A) or severe (F31A, Y229A) defects (Fig. 6; Table S4). Interestingly, V99A parallels a known activating mutation in *B. subtilis* GerAB (37), reinforcing its functional significance. Notably, all mutant spores assembled visible germinosomes, indicating that germination defects reflect impaired function, not their interaction with other GR subunits.

### Cooperativity between GerI and GerQ

**Fig 7:**
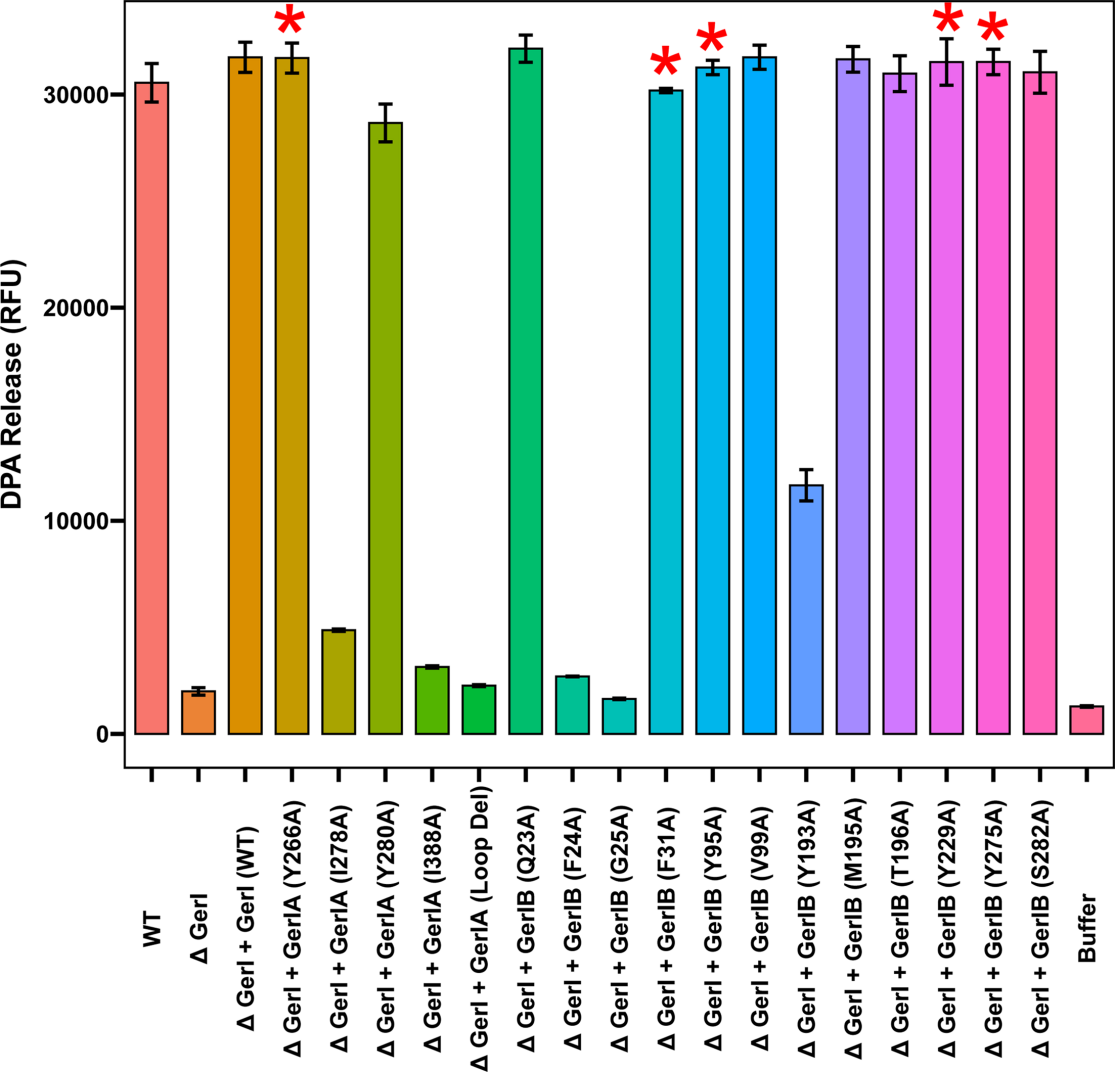
DPA release by *B. cereus gerI* spores ectopically expressing plasmid-borne GerIA and GerIB variants. Spores were heat-shocked (75°C for 30 min) and cooled before resuspending in buffer (50 mM Tris-HCl, 100 mM NaCl, pH 8.0, supplemented with 1 mM inosine and 50 µM TbCl_3_). Germination was monitored using fluorimetric measurements of DPA release as described in the Materials and Methods in the supplemental material. Presented mean values are endpoint measurements taken after 3 h and derived from experiments conducted in triplicate with single batches of spores. Error bars represent SD from the mean. The red asterisks indicate variant strains with impaired germination in the *gerI gerQ* background, as shown in Fig. 4B and 6.

Having identified key GerIA and GerIB residues in GerI GR function, we next asked whether GerQ, which is largely inactive in the absence of GerI (Fig. 4A), might complement or influence germination defects in strains expressing variant GerI GRs. Accordingly, plasmids encoding GerI variants were introduced into the *B. cereus gerI* null strain, and the resultant spores were assessed for inosine-induced germination and the presence of GerI-associated germinosomes. DPA release assays revealed that germination efficiency was restored to near wild-type levels in nine of the twelve GerIB variant strains tested, including some with essentially zero (Y229A) or significantly impaired (F31A, Y95A, Y275A) germination in the *gerI gerQ* null background (Fig. 7; Fig. S5). However, germination remained minimal (<5% DPA release) in two GerIB variant strains (F24A and G25A), both in the predicted unwound region of TM1. Partial restoration was observed in the GerIB Y193A variant, with ~30% DPA release.

A similar trend was observed with GerIA variants: Y266A and Y280A spores germinated efficiently and formed visible germinosomes, while I278A and I338A showed poor germination (~10%–15% DPA release) and lacked detectable germinosomes (Fig. 7; Fig. S5 and S6). Notably, spores with the GerIA N1–N2 loop deletion retained germinosomes but failed to germinate (Fig. 7; Fig. S6). Together, these findings suggest that GerQ can partially compensate for certain GerI mutations, and that GerIA clustering is a prerequisite for inosine germination, at least based on the modest number of residues tested here. Equally, assembly of GerIA into germinosomes is not necessarily commensurate with inosine-triggered germination, regardless of whether mutations are to the A or B subunits of the GR.

### Comparison of germinant binding in GerIB and GerQB

Initial studies conducted with *B. cereus* 569 (ATCC 10876) spores bearing transposon insertions at loci encoding GerI and GerQ indicated that both GRs are required for germination with inosine as a sole germinant (21, 24). However, experiments conducted in the present study indicate that inosine-mediated germination can be initiated by GerI in *gerQ* null spores, and this is faster when the GR is expressed from a low copy number ectopic plasmid compared to the chromosomal locus (Fig. 4A). However, only very modest inosine germinative responses are evident in spores with chromosomal (<5%) or plasmid (<10%) expressed GerQ that are null for *gerI*. A number of scenarios could account for apparent differences in GerI and GerQ sensitivity toward inosine, including postulated structural disparities in germinant and co-germinant binding sites (25, 26). Despite sharing only 38% amino acid sequence identity, AlphaFold structures of GerIB and GerQB superimpose with an RMSD of 0.83 Å for 325 Cα pairs, hence their overall predicted folds are extremely similar (Fig. S7). However, seven of the twelve residues identified as contributing to the GerIB inosine and Na^+^ binding pockets are either conservatively substituted or not conserved in GerQB (Table S4), including in two positions (F24M and Y193F) where the presence of GerQ failed to complement inosine germination in spores with hypomorphic *gerI* alleles (Fig. 7).

In the absence of experimental structural data, Chai-1 predictions may be informative concerning binding distinctions between the two proteins. When presented collectively with inosine, L-alanine, and a single Na^+^ ion, for example, the algorithm predicts binding locations within GerQB that are analogous to those described previously for GerIB (Fig. 5; Fig. S7). In these models, L-alanine is located in a position equivalent to that observed in the co-crystal structure of the GkApcT–L-alanine complex (hereafter referred to as Site 1), with Na^+^ in a pocket equivalent to the LeuT Na^+^2 pocket (Site 2). Inosine occupies a pocket (Site 3) that sits above L-alanine binding Site 1, with both pockets occupying an exterior-facing solvent-accessible channel (Fig. S7). However, when presented with GerIB plus only inosine and Na^+^, the algorithm places inosine at Site 1 while Na^+^ remains unchanged at Site 2. Conversely, in GerQB, the Na^+^ ion occupies Site 1 while inosine is located at Site 3. L-alanine is consistently placed at Site 1 regardless of whether Na^+^ and/or inosine is present in both GerIB and GerQB. Cautious interpretation of these models, which, as before, have high confidence metrics associated with the overall predicted structures but are less assured in terms of ligand placements, points to a potential scenario where productive binding of inosine at Site 1 occurs only in GerIB and not in GerQB, and both GR subunits preferentially bind L-alanine at this position. Whether the latter is sufficient to trigger germination is not clear and will necessitate further work with these GRs in strains null for all other GRs. It is also notable that Chai-1 predicts binding at Site 1 for amino acids that do not initiate germination, such as L-valine and L-leucine, so while these amino acids may well be cognate but bind unproductively, inferences drawn from the various models should be tempered accordingly.

## DISCUSSION

The introduction to this paper alludes to an incomplete picture of spore GR structure and function. In the absence of experimental structures of assembled GR complexes captured at various stages of activation, this remains the case. Regardless, the present work has made significant contributions in terms of insight to the *B. cereus* inosine-responsive GRs. Notably, the crystal structure of GerIA^NTD^ represents only the third experimental structure of a spore GR subunit, or a significant fragment thereof. That the latter’s fold is similar to that previously revealed for the equivalent fragment of the *B. megaterium* GerK_3_A subunit is perhaps not surprising but adds a layer of experimental validation to the suggestion that equivalent subunits across the spore GR family probably adopt similar folds and, by extension, quaternary organization and function. GerIA^NTD^ is revealed to be predominantly monomeric in aqueous solution, which is different from the predicted oligomerization state of the full-length protein in the favored AlphaFold pentameric or hexameric models. The same discrepancy applies to the crystal structure of GerK_3_A^NTD^, which is a monomer, and to the GerBC crystal structure, where an interlocked dimer is evident within the unit cell but the protein is monomeric in solution. However, given the confidence metrics associated with the AlphaFold model, and associated evolutionary coupling, cross-linking, and mutagenesis analyses (14, 15), it seems highly likely that the membrane-spanning components of the A subunits promote GR complex assembly.

The current study additionally confers insight to the sensing of inosine as a germinant in *B. cereus* spores. In the first instance, recombinant GerIA^NTD^ was shown by NMR CSP analyses to be responsive to inosine, albeit at considerably higher concentrations than those that stimulate efficient spore germination (40 mM versus 1 mM). The physiological significance of the GerIA^NTD^ response to inosine has not been established—indeed, perhaps other germinants elicit similar chemical shifts—but mutagenesis experiments have revealed the functional and/or structural importance of most of the residues and subdomain linker in question. However, consistent with previous studies (14), our proteinase K accessibility assays using germinated, decoated *B. subtilis* spores support a model that places the *B. subtilis* GerAA^NTD^ and presumably all A-NTDs firmly within the spore core (Fig. S8; supplemental results). This localization makes it highly unlikely that this region of the A subunits is directly involved in the initial sensing of germinants. Indeed, the core location is more commensurate with a role for the A-NTD in gating or facilitating ion transit from the core prior to efflux through the channel formed by oligomeric A-subunit membrane domains. Notably, the structural and/or functional significance of A-NTD is underscored by the identification of *B. subtilis* GerAA^NTD^ residues that suppress the hypermorphic GerAA P326A allele (the latter being localized in the GerAA membrane domain) (38) (Fig. S9), suggesting that the A-NTD serves to stabilize the GR, perhaps by anchoring the complex within the spore core.

Elsewhere, AlphaFold models superposed with ligand-bound crystal structures of APC transporters further substantiate the idea that GR B-subunit proteins constitute the germinant binding site in spores (37). This approach identified a putative pocket within the GerIB protein that can accommodate an intact inosine molecule at a position analogous to that observed for L*-*alanine in the *B. subtilis* GerAB protein (14). It’s noteworthy that an intact inosine molecule can be accommodated since inosine is significantly larger than most common germinants and enzymatic degradation of purine ribosides by an exosporium-located hydrolase has been recognized for many decades (39), although neither ribose nor the hypoxanthine base individually triggers germination (Fig. S10). Binding of a Na^+^ ion at a GerIB location equivalent to the LeuT Na^+^2 site is also plausible and supported by SDM data that generally demonstrates impaired GerI function when candidate ligand binding residues are substituted. However, the question as to whether inosine and L*-*alanine can bind concurrently to GerIB—and which may underpin co-germinant synergy—remains open since neither superposed crystal structures nor Chai-1 models unambiguously or confidently predict binding of both germinants at the same time. Indeed, the Chai-1 algorithm predicted a second potential binding site for inosine and additionally predicted differences in germinant and co-germinant binding patterns between GerIB and GerQB that may underpin functional differences between the receptors. While it should be possible to test associated hypotheses *in vivo* with appropriate mutant constructs, it seems likely that high-resolution experimental structures with bound ligands will be required to confer deeper insight into the precise locale of binding sites and potential conformational changes that occur within B-subunit proteins. Interestingly, our proteinase K protection assays also show that *B. subtilis* GerBC protein is resistant to digestion (Fig. S8; supplemental results), a finding that appears inconsistent with its predicted outer IM location by AlphaFold and may instead reflect shielding by other components of the IM or structural features of the dormant spore. Clearly, this observation, together with the mechanisms underlying GR complex assembly, warrants further investigation.

The basis of the cooperativity observed in the current study between GRs, where GerQ could complement hypomorphic *gerIA* and *gerIB* alleles, is also not clear. It’s worth noting that GerQ can form germinosomes in the *gerI* null background (Fig. S6F), so GerQ has no obvious dependency on GerI for localization within the spore. That being the case, a number of alternative options could account for the observed GerQ-mediated complementation, including (i) assembly of hybrid (IA-QB or QA-IB) protomers, (ii) assembly of GerI/GerQ hetero-oligomer GR complexes, or (iii) functional GerQ requires GerI incorporation into the germinosome, even if the GerI variant itself is non-functional.

How then do we integrate findings from the current work into contemporary models of inosine-mediated spore germination in *B. cereus*? The simplest model entails diffusion of inosine through the outer layers of the spore to bind at one of two potential sites within the GerIB protein. Binding induces an allosteric interaction with an adjacent GerIA subunit, in a process that is facilitated by co-binding of a Na^+^ ion within GerIB, and which ultimately leads to opening of the GerIA central ion channel. This model, which is analogous to that proposed on the basis of molecular genetic evidence for the *B. subtilis* GerA alanine-responsive GR (38), abrogates both the physiological significance of GerIA^NTD^ CSPs to inosine and structural similarities between A-NTDs and their ABC transporter substrate-binding-domain counterparts. The same model can be applied to the GerQ GR, where inosine, L-alanine, and Na^+^ occupy their designated binding pockets within GerQB, and where presumably the binding energy associated with primary and co-germinant(s) interaction with the receptor is necessary to drive the allosteric response. An alternative model that considers GerIA^NTD^ inosine responsiveness could entail the transport of inosine across the membrane by GerIB via mechanisms and movements that are employed by distantly related APC transporters (40). Transported inosine could then diffuse and interact with GerIA^NTD^ at potential ligand-binding sites identified in this work, and then allosterically stimulate opening of the central ion channel. However, in the absence of an obvious ion gradient or other energy source to drive germinant transport across the membrane, the simpler allosteric model is perhaps more likely. Clarification of models that describe the earliest stages of spore germination and subsequent transduction of these signals to downstream components of the germination apparatus remains the focus of work in this area.

## MATERIALS AND METHODS

Detailed experimental procedures are provided in the Supplemental material. Briefly, proteins were expressed, purified, and crystallized by using standard methods. X-ray diffraction data were collected at the Stanford Synchrotron Radiation Light Source (SSRL) and National Synchrotron Light Source II (NSLS-II). The GerIA^NTD^ structure was determined using MAD phasing. Mutant strains of *B. cereus* were constructed by allelic exchange, using plasmid-borne variants of GerI with GerIA-GFP for complementation-type analyses.

## Data Availability

The atomic coordinates and structure factors of GerIA^NTD^ have been deposited in the Protein Data Bank (https://www.wwpdb.org/) with PDB ID code 9PRB.

## SUPPLEMENTAL MATERIAL

Supplemental material mbio.00108-26-s0001.docx Supplemental results and methods, Tables S1 to S4, and Fig. S1 to S10.

ASM does not own the copyrights to Supplemental Material that may be linked to, or accessed through, an article. The authors have granted ASM a non-exclusive, world-wide license to publish the Supplemental Material files. Please contact the corresponding author directly for reuse.

## References

[1] Setlow P, Christie G. 2023. New thoughts on an old topic: secrets of bacterial spore resistance slowly being revealed. Microbiol Mol Biol Rev 87:e00080-22. doi:10.1128/mmbr.00080-22 36927044 PMC10304885

[2] Setlow P, Johnson EA. 2019. Spores and their significance, p 23–64. In Doyle MP, Buchanan R (ed), Food microbiology, fundamentals and frontiers, 5th ed. ASM Press, Washington DC.

[3] Christie G, Setlow P. 2020. Bacillus spore germination: Knowns, unknowns and what we need to learn. Cell Signal 74:109729. doi:10.1016/j.cellsig.2020.109729 32721540

[4] Lyu F, Zhang T, Gui M, Wang Y, Zhao L, Wu X, Rao L, Liao X. 2023. The underlying mechanism of bacterial spore germination: an update review. Compr Rev Food Sci Food Saf 22:2728–2746. doi:10.1111/1541-4337.13160 37125461

[5] Atluri S, Ragkousi K, Cortezzo DE, Setlow P. 2006. Cooperativity between different nutrient receptors in germination of spores of Bacillus subtilis and reduction of this cooperativity by alterations in the GerB receptor. J Bacteriol 188:28–36. doi:10.1128/JB.188.1.28-36.2006 16352818 PMC1317597

[6] Zuberi AR, Feavers IM, Moir A. 1985. Identification of three complementation units in the gerA spore germination locus of Bacillus subtilis. J Bacteriol 162:756–762. doi:10.1128/jb.162.2.756-762.1985 2985546 PMC218915

[7] Paidhungat M, Setlow P. 2000. Role of ger proteins in nutrient and nonnutrient triggering of spore germination in Bacillus subtilis. J Bacteriol 182:2513–2519. doi:10.1128/JB.182.9.2513-2519.2000 10762253 PMC111315

[8] Hudson KD, Corfe BM, Kemp EH, Feavers IM, Coote PJ, Moir A. 2001. Localization of GerAA and GerAC germination proteins in the Bacillus subtilis spore. J Bacteriol 183:4317–4322. doi:10.1128/JB.183.14.4317-4322.2001 11418573 PMC95322

[9] Paidhungat M, Setlow P. 2001. Localization of a germinant receptor protein (GerBA) to the inner membrane of Bacillus subtilis spores. J Bacteriol 183:3982–3990. doi:10.1128/JB.183.13.3982-3990.2001 11395462 PMC95281

[10] Ramirez-Peralta A, Gupta S, Butzin XY, Setlow B, Korza G, Leyva-Vazquez MA, Christie G, Setlow P. 2013. Identification of new proteins that modulate the germination of spores of Bacillus species. J Bacteriol 195:3009–3021. doi:10.1128/JB.00257-13 23625846 PMC3697528

[11] Griffiths KK, Zhang J, Cowan AE, Yu J, Setlow P. 2011. Germination proteins in the inner membrane of dormant Bacillus subtilis spores colocalize in a discrete cluster. Mol Microbiol 81:1061–1077. doi:10.1111/j.1365-2958.2011.07753.x 21696470 PMC7959159

[12] Wang Yan, de Boer R, Vischer N, van Haastrecht P, Setlow P, Brul S. 2020. Visualization of germination proteins in putative Bacillus cereus germinosomes. IJMS 21:5198. doi:10.3390/ijms21155198 32707970 PMC7432890

[13] Wang Y, Breedijk RMP, Hink MA, Bults L, Vischer NOE, Setlow P, Brul S. 2021. Dynamics of germinosome formation and FRET-based analysis of interactions between GerD and germinant receptor subunits in Bacillus cereus spores. IJMS 22:11230. doi:10.3390/ijms222011230 34681888 PMC8539644

[14] Gao Y, Amon JD, Artzi L, Ramírez-Guadiana FH, Brock KP, Cofsky JC, Marks DS, Kruse AC, Rudner DZ. 2023. Bacterial spore germination receptors are nutrient-gated ion channels. Science 380:387–391. doi:10.1126/science.adg9829 37104613 PMC11154005

[15] Kilian M, Bischofs IB. 2023. Co-evolution at protein-protein interfaces guides inference of stoichiometry of oligomeric protein complexes by de novo structure prediction. Mol Microbiol 120:763–782. doi:10.1111/mmi.15169 37777474

[16] Wilson MJ, Carlson PE, Janes BK, Hanna PC. 2012. Membrane topology of the Bacillus anthracis GerH germinant receptor proteins. J Bacteriol 194:1369–1377. doi:10.1128/JB.06538-11 22178966 PMC3294866

[17] Korza G, Setlow P. 2013. Topology and accessibility of germination proteins in the Bacillus subtilis spore inner membrane. J Bacteriol 195:1484–1491. doi:10.1128/JB.02262-12 23335419 PMC3624538

[18] Li Y, Setlow B, Setlow P, Hao B. 2010. Crystal structure of the GerBC component of a Bacillus subtilis spore germinant receptor. J Mol Biol 402:8–16. doi:10.1016/j.jmb.2010.07.018 20654628 PMC3607951

[19] Li Y, Jin K, Perez-Valdespino A, Federkiewicz K, Davis A, Maciejewski MW, Setlow P, Hao B. 2019. Structural and functional analyses of the N-terminal domain of the A subunit of a Bacillus megaterium spore germinant receptor. Proc Natl Acad Sci USA 116:11470–11479. doi:10.1073/pnas.1903675116 31113879 PMC6561283

[20] Pustovalova Y, Li Y, Hoch JC, Hao B. 2025. Backbone assignment of the N-terminal domain of the A subunit of the Bacillus cereus GerI germinant receptor. Biomol NMR Assign 19:47–52. doi:10.1007/s12104-025-10216-7 39826051

[21] Clements MO, Moir A. 1998. Role of the gerI operon of Bacillus cereus 569 in the response of spores to germinants. J Bacteriol 180:6729–6735. doi:10.1128/JB.180.24.6729-6735.1998 9852021 PMC107780

[22] Weiner MA, Read TD, Hanna PC. 2003. Identification and characterization of the gerH operon of Bacillus anthracis endospores: a differential role for purine nucleosides in germination. J Bacteriol 185:1462–1464. doi:10.1128/JB.185.4.1462-1464.2003 12562819 PMC142867

[23] Ireland JAW, Hanna PC. 2002. Amino acid- and purine ribonucleoside-induced germination of Bacillus anthracis DeltaSterne endospores: gerS mediates responses to aromatic ring structures. J Bacteriol 184:1296–1303. doi:10.1128/JB.184.5.1296-1303.2002 11844758 PMC134857

[24] Barlass PJ, Houston CW, Clements MO, Moir A. 2002. Germination of Bacillus cereus spores in response to L-alanine and to inosine: the roles of gerL and gerQ operons. Microbiology (Reading) 148:2089–2095. doi:10.1099/00221287-148-7-2089 12101297

[25] Abel-Santos E, Dodatko T. 2007. Differential nucleoside recognition during Bacillus cereus 569 (ATCC 10876) spore germination. New J Chem 31:748. doi:10.1039/b616695d

[26] Dodatko T, Akoachere M, Jimenez N, Alvarez Z, Abel-Santos E. 2010. Dissecting interactions between nucleosides and germination receptors in Bacillus cereus 569 spores. Microbiology (Reading) 156:1244–1255. doi:10.1099/mic.0.030270-0 20035009 PMC2889443

[27] Hornstra LM, de Vries YP, Wells-Bennik MHJ, de Vos WM, Abee T. 2006. Characterization of germination receptors of Bacillus cereus ATCC 14579. Appl Environ Microbiol 72:44–53. doi:10.1128/AEM.72.1.44-53.2006 16391023 PMC1352193

[28] Schuck P. 2000. Size-distribution analysis of macromolecules by sedimentation velocity ultracentrifugation and lamm equation modeling. Biophys J 78:1606–1619. doi:10.1016/S0006-3495(00)76713-0 10692345 PMC1300758

[29] Evans R, O’Neill M, Pritzel A, Antropova N, Senior A, Green T, Žídek A, Bates R, Blackwell S, Yim J, Ronneberger O, Bodenstein S, Zielinski M, Bridgland A, Potapenko A, Cowie A, Tunyasuvunakool K, Jain R, Clancy E, Kohli P, Jumper J, Hassabis D. 2022. Protein complex prediction with AlphaFold-Multimer. bioRxiv. doi:10.1101/2021.10.04.463034:2021.10.04.463034

[30] Jumper J, Evans R, Pritzel A, Green T, Figurnov M, Ronneberger O, Tunyasuvunakool K, Bates R, Žídek A, Potapenko A, et al.. 2021. Highly accurate protein structure prediction with AlphaFold. Nature 596:583–589. doi:10.1038/s41586-021-03819-2 34265844 PMC8371605

[31] Abramson J, Adler J, Dunger J, Evans R, Green T, Pritzel A, Ronneberger O, Willmore L, Ballard AJ, Bambrick J, et al.. 2024. Accurate structure prediction of biomolecular interactions with AlphaFold 3. Nature 630:493–500. doi:10.1038/s41586-024-07487-w 38718835 PMC11168924

[32] Suzuki S, Henderson PJF. 2006. The hydantoin transport protein from Microbacterium liquefaciens. J Bacteriol 188:3329–3336. doi:10.1128/JB.188.9.3329-3336.2006 16621827 PMC1447452

[33] Weyand S, Shimamura T, Yajima S, Suzuki S, Mirza O, Krusong K, Carpenter EP, Rutherford NG, Hadden JM, O’Reilly J, Ma P, Saidijam M, Patching SG, Hope RJ, Norbertczak HT, Roach PCJ, Iwata S, Henderson PJF, Cameron AD. 2008. Structure and molecular mechanism of a nucleobase-cation-symport-1 family transporter. Science 322:709–713. doi:10.1126/science.1164440 18927357 PMC2885439

[34] Simmons KJ, Jackson SM, Brueckner F, Patching SG, Beckstein O, Ivanova E, Geng T, Weyand S, Drew D, Lanigan J, Sharples DJ, Sansom MSP, Iwata S, Fishwick CWG, Johnson AP, Cameron AD, Henderson PJF. 2014. Molecular mechanism of ligand recognition by membrane transport protein, Mhp1. EMBO J 33:1831–1844. doi:10.15252/embj.201387557 24952894 PMC4195764

[35] Jungnickel KEJ, Parker JL, Newstead S. 2018. Structural basis for amino acid transport by the CAT family of SLC7 transporters. Nat Commun 9:550. doi:10.1038/s41467-018-03066-6 29416041 PMC5803215

[36] Boitreaud J, Dent J, McPartlon M, Meier J, Reis V, Rogozhnikov A, Wu K. 2024. Chai-1: Decoding the molecular interactions of life. bioRxiv. doi:10.1101/2024.10.10.615955:2024.10.10.615955

[37] Artzi L, Alon A, Brock KP, Green AG, Tam A, Ramírez-Guadiana FH, Marks D, Kruse A, Rudner DZ. 2021. Dormant spores sense amino acids through the B subunits of their germination receptors. Nat Commun 12:6842. doi:10.1038/s41467-021-27235-2 34824238 PMC8617281

[38] Amon JD, Artzi L, Rudner DZ. 2022. Genetic evidence for signal transduction within the Bacillus subtilis GerA germinant receptor. J Bacteriol 204:e0047021. doi:10.1128/JB.00470-21 34780301 PMC8846391

[39] Powell JF. 1957. Biochemical changes occurring during spore germination in Bacillus species. J Appl Bacteriol 20:349–358. doi:10.1042/bj0630661

[40] Beckstein O, Naughton F. 2022. General principles of secondary active transporter function. Biophys Rev (Melville) 3:011307. doi:10.1063/5.0047967 35434715 PMC8984959

