# Supplementary material for: Structural and functional analysis of the Bacillus cereus GerI inosine-responsive spore germinant receptor
Source: mBio. 2026 Mar 27;17(5):e00108-26. doi: 10.1128/mbio.00108-26 (PMC13170314; doi:10.1128/mbio.00108-26)
Supplement: Supplemental material — Supplemental results and methods, Tables S1 to S4, and Fig. S1 to S10. [file mbio.00108-26-s0001.docx]

**SUPPLEMENTAL MATERIAL**

**Title:** Structural and functional analysis of the *Bacillus cereus* GerI inosine-responsive spore germinant receptor

**Author affiliation:**  ^1*^Yunfeng Li, ^2*^Giannina Ow-Young-Villarreal, ^1^Yulia Pustovalova, ^3^David M.D. Bailey, ^2^Joshua Yarrow, ^1^George Korza, ^1^Faith Ye, ^4^Heidi Erlandsen, ^1^Peter Setlow, ^2#^Graham Christie and ^1#^Bing Hao

^1^Department of Molecular Biology and Biophysics, UConn Health, Farmington, CT 06030, USA; ^2^Department of Chemical Engineering and Biotechnology, University of Cambridge, Cambridge, United Kingdom; ^3^Department of Zoology, University of Cambridge, Cambridge, United Kingdom; and ^4^Department of Pharmaceutical Science, University of Connecticut, Storrs, CT 06269, USA

^#^ Correspondence to Graham Christie (gc301@cam.ac.uk) and Bing Hao (bhao@uchc.edu)

* Yunfeng Li and Giannina Ow-Young-Villarreal contributed equally to this work

**SM Appendix List**

1. SM Results
2. SM Materials and Methods
3. SM Tables
4. SM Figures and Figure Legends
5. SM References

**SM RESULTS**

**Proteinase K accessibility of GR A and C subunits**

To detect exogenous germinants, any GR subunit must be exposed to the spore’s external environment. Previous studies using proteinase K protection assays on protoplasts isolated from vegetatively growing *B. subtilis* cells expressing GerA GR subunits suggested that the GerAA NTD resides in the spore core (1). To clarify the membrane topology of GR A and C-subunits in spores, and in the absence of GerI-specific antisera, we examined the proteinase K sensitivity of endogenous GerAA and GerBC in germinated, decoated *B. subtilis* spores. Proteinase K is expected to digest protein regions exposed on the outer IM surface but not those facing the spore core. After 40 min of proteinase K treatment, a ~25 kDa GerAA fragment remained resistant to further digestion (Fig. S8A). Since anti-GerAA antibodies were raised against its soluble NTD, which is smaller than the protected fragment, this suggests the cleavage likely occurred in an extracellular C-terminal loop within the transmembrane domain of GerAA. In contrast, GerBC was largely resistant to digestion (Fig. S8A), despite being readily cleaved in purified form lacking its signal and lipobox sequences (Fig. S8B).

While supporting earlier results (2, 3) and the hypothesis that GR A-NTDs are core-localized, the basis for intact C protein persistence after proteinase treatment is not obvious. The presence of N-terminal signal and lipobox sequences associated with GR C-subunits strongly indicates translocation across IM from the forespore and Lgt-catalyzed anchoring to the outer IM surface (4, 5). Similarly, of the various iterations of GR assemblies predicted by AlphaFold, none are consistent with placement of C-subunits on the same side of the membrane as the hydrophilic A-NTDs (data not shown). Regardless of the hypothetical scenarios, GR C-subunits appear largely inaccessible in germinated coat-compromised spores to chemical labelling (3) and protease digestion (2). One possible explanation for this apparent anomaly is that the C-subunits are indeed located on the outer surface of the IM but are somehow rendered inaccessible to labelling reagents and proteases by the surface conformation of the IM, or by other proteins present in the germinosome. The GerD lipoprotein, which appears to have a scaffold-like function and structure in germinosome assembly (6-8) and possesses signal and lipobox motifs similar to those of GR C-subunits, is an obvious candidate for such a protective role. However, GerD is rapidly degraded in germinated spores exposed to proteinase K (Fig. S8A), consistent with its assumed location on the outer IM surface. Unfortunately, neither the precise architecture nor protein inventory of germinosomes is well understood, and it may be that candidate lipoproteins, such as members of the YhcN or YetF-type proteins (9, 10), have a role in shielding GR C-subunits from potentially hazardous chemicals and enzymes in spores. Evidently, this will require further investigation of the functions of GRs and other components of spore IM as a whole.

**SM MATERIALS AND METHODS**

**Protein expression and purification**

Genes encoding *gerIA* (Biocyc ID EJ379_RS24150; *B. cereus* strain ATCC 14579), *gerAA* (Biocyc ID BSU33050; *B. subtilis* strain PS832), *gerBC* (Biocyc ID BSU35820), and *gerD* (Biocyc ID BSU01550) were PCR-amplified and cloned into a modified pET15b vector with an N-terminal removable tobacco etch virus (TEV) protease-cleavable His_6_ tag. Truncated constructs, GerIA^NTD^ (residues 245-500; 29.4 kDa), GerIA^NTD2^ (238-484; 28.8 kDa), GerAA^NTD^ (1-239; 27.5 kDa), GerBC (25-374; 40.1 kDa), and GerD (27-185; 18.6 kDa), were expressed in *Escherichia coli* BL21(DE3) and purified by Ni^2+^-nitriloacetic acid (GE Healthcare) affinity chromatography. Following TEV cleavage, proteins were further purified by ion exchange (Source Q or S) and size exclusion chromatography (SD200; GE Healthcare). For crystallization, GerIA^NTD^ was concentrated to 0.69 mM in 15 mM Tris-HCl (pH 7.6), 150 mM NaCl, and 5 mM dithiothreitol (DTT); the GerAA^NTD2^, GerBC, and GerD proteins were concentrated and stored in the same buffer. SeMet-substituted GerIA^NTD^ was produced following established procedures (11) and purified as described above. For NMR, ^15^N-labeled GerIA^NTD2^ was expressed in M9 media supplemented with ^15^NH_4_Cl (1 g/L, Cambridge Isotope Laboratories) as the sole nitrogen source and purified as described above. The ^15^N-labeled GerIA^NTD2^ was concentrated to 0.45 mM in buffer containing 20 mM Na/K phosphate (pH 7.2), 150 mM NaCl, 2 mM β-mercaptoethanol, 8% D_2_O, and 0.02% NaN_3_, immediately prior to data acquisition.

**Crystallization and structure determination**

GerIA^NTD^ was crystallized at 16°C by hanging-drop vapor diffusion in 0.1 M HEPES (pH 7.4), 1.8-2.0 M ammonium acetate. Crystals were cryoprotected with reservoir solution supplemented with 10% glycerol and flash-cooled in liquid nitrogen. X-ray diffraction data were collected at NSLS-II beamlines 17-ID-1 and 17-ID-2 and SSRL beamline 9-2, and processed using Fast DP (12, 13). The crystals contain three molecules in the asymmetric unit. The structure of SeMet-substituted GerIA^NTD^ was determined by MAD using peak and inflection data. About 27 selenium sites were located by HySS (14) as implemented in AutoSol/PHENIX (15), and initial phases were improved with Phaser/PHENIX (16). Over 90% of the model was built in COOT (17) using the resulting electron density map, followed by refinement with REFMAC5/CCP4 (18, 19), BUSTER (Global Phasing Limited) followed and manual rebuilding. Data collection, phasing, and refinement statistics are summarized in Table S1. Ramachandran statistics were calculated using MolProbity (20), and figures generated in PyMOL (Schrödinger LLC).

**NMR chemical shift mapping**

Two-dimensional ^1^H–^15^N HSQC spectra of the ^15^N-labeled GerIA^NTD2^ were collected at 25°C on an Agilent VNMRS 800 MHz spectrometer with an HCN cryoprobe (21). For titration, GerIA^NTD2^ (final concentration 0.25 mM) was mixed with inosine (40 mM) prepared in matching buffer composition and pH. Spectra for apo and ligand-bound states were acquired under identical conditions. All spectra were processed with NMRPipe (22), and analyzed using CcpNmr Analysis (23) via NMRbox (24). CSPs ($\Delta\delta$) were calculated by using the following equation (25):

$$\Delta\delta=\sqrt{[\Delta\delta_{H}^{2}+{(\alpha\cdot{\Delta\delta}_{N})}^{2}]}$$

where ${\Delta\delta}_{H}$ and ${\Delta\delta}_{N}$ are the ^1^H and ^15^N chemical shift differences, respectively, and the scaling factor α was set to 0.154 (26), based on BioMagResBank statistics (27).

***Bacillus cereus* strains, culture, and spore preparation**

*B. cereus* strains employed in this study (Table S3) are isogenic with the ATCC 10876 strain. ATCC 10876 was selected for site-directed mutagenesis (SDM) studies because it sporulates more efficiently and produces spores that germinate faster and more consistently than those of ATCC 14579. Cultures were grown in LB medium at 37°C with antibiotics as required. *E. coli* Turbo and dam^-^/dcm^-^ strains (New England Biolabs, UK) were used for cloning and plasmid propagation. *B. cereus* spores were prepared by nutrient exhaustion in Supplemented Nutrient Broth (SNB) medium. Sporulation was achieved by spreading 1 ml of overnight culture on SNB agar plates and incubating at 30°C for 72 hrs. Spores were harvested, washed repeatedly in ice-cold water, and purified by centrifugation until >99% phase bright spores were confirmed by phase contrast microscopy. Purified spores were stored in water at an OD_600_ of approximately 50 at 4°C.

**Molecular biology procedures for *Bacillus cereus***

*B. cereus* *gerI*, *gerQ*, and *gerI gerQ* null mutant strains were prepared using the markerless allelic exchange procedure described previously (28, 29), leaving only the first and last codons of each operon. Mutations were confirmed by DNA sequencing. To construct the *B. cereus* ATCC 14579 *gerIA-gfp* fusion strain, PCR was used to generate a *gerI* promoter-*gerIA-gfp* amplicon, where the *gfp* ORF was located in-frame at the 3’end of the *gerIA* ORF. A second PCR amplicon encompassing the downstream *gerIB*, *gerIC* and *gerID* ORFs was prepared and then the various fragments were cloned into the low-copy pHT304 plasmid (30) to reconstitute the full operon. Similar procedures were used to make the equivalent *B. cereus* ATCC 14579 *gerQ* operon including *gerQA-gfp*. SDM was performed using the QuikChange Lightning Kit (Agilent Technologies, UK), and all constructs were verified by sequencing.

**Spore germination** **for *Bacillus cereus***

*B. cereus* spore germination was monitored using a terbium-DPA fluorescence assay (31). Heat-shocked spores (75°C, 30 min) were cooled on ice and resuspended (OD_600_ = 1.0) in 100 µL buffer (50 mM Tris-HCl, 100 mM NaCl, pH 8.0) supplemented with 1 mM inosine and 50 µM TbCl_3_ (Sigma Aldrich) in black 96-well plates. Fluorescence (Ex: 337 nm; Em: 620 nm) was monitored over 3 hrs at 30°C using a BMG Labtech CLARIOstar Microplate Reader operating in homogeneous time-resolved fluorescence mode with orbital shaking. Data shown are from representative experiments with <15% standard deviation (SD) across at least three independent spore batches. Germination was also assessed by OD_600_ loss and by phase contrast microscopy.

***Bacillus subtilis* strains, spore preparation, purification, and germination**

*B. subtilis* strain PS4150, an isogenic derivative of PS832 (from strain 168), carries Δ*cotE*::tet and Δ*gerE*::spc deletions affecting spore coat assembly (32). Strains were grown in LB medium at 37°C, and spores were prepared on 2 x SG agar plates, harvested and purified as described (33, 34). The purified spores were >98% pure by phase-contrast microscopy and stored in the dark at 4°C. Prior to germination, PS832 spores were decoated at 70°C for 2 hrs in 0.1 M NaOH, 0.1 M NaCl, 1% SDS, and 0.1 M β-mercaptoethanol, then washed extensively with water (3). PS4150 spores were heat activated at 70°C in water for 1 hr. Germination was conducted at 37°C using heat-activated spores (OD_600_ = 50 for PS4150; 100 for PS832) in 25 mM sodium HEPES (pH 7.4) with 10 mM each of L*-*valine, L*-*asparagine, glucose, fructose, and KCl. Spores were incubated for 2 hrs (PS4150) or 4 hrs (PS832) until > 90% of spores were germinated, confirmed by phase-contrast microscopy, immediately prior to proteinase K accessibility assays.

**Microscopy**

Samples were applied to poly-L*-*lysine-coated microscope slides, covered with coverslips, and imaged using an Olympus BX53 microscope with a 100× 1.30 numerical aperture (NA) oil objective lens and GFP filters under mercury lamp illumination. Images were captured with a Retiga-2000R CCD camera (74 nm/pixel, 12-bit. 1600 x 1200 Tiff format) and processed using Adobe Photoshop.

**Analytical ultracentrifugation**

Sedimentation velocity measurements were performed at 42,000 rpm at 20°C using a Beckman-Coulter Optima analytical ultracentrifuge with an An-60 Ti rotor. GerIA^NTD^ was run on a gel filtration column (SD75 increase; GE Healthcare) in 15 mM Tris-HCl (pH 7.6), 150 mM NaCl, and 2 mM DTT, and analyzed at 10 and 32 µM in two-channel aluminum-Epon double-sector centerpieces with sapphire windows. Absorbance at 280 nm was recorded every 20s for ~12 hrs. Data were analyzed by the *c*(s) method using SEDFIT (35). Solvent density and protein partial specific volume were estimated using SEDNTERP (36).

**Proteinase K accessibility assay**

Germinated PS4150 spores (OD_600_ = 50) and decoated, germinated PS832 spores (OD_600_ = 100, sonicated three min in a water bath) were washed and resuspended in 50 mM Tris-HCl (pH 7.6), then each split into six aliquots. Proteinase K (Invitrogen) was added to five aliquots at 50 µg/mL (PS4150) or 83 µg/mL (PS832) and incubated at 30°C or 37°C, respectively, for 0, 5, 10, 20 or 40 min. The zero-minute time point was used to confirm proteinase neutralization by comparing it with the sixth aliquot, which lacks the proteinase and serves as a control. Digestion was terminated with 5 mM phenylmethylsulfonyl fluoride (PMSF), and samples were placed on ice. After adding the Laemmli sample loading dye, the samples were heated at 60°C for 15 min and analyzed by SDS-PAGE and Western blot using equal amounts of the digested samples against specific rabbit polyclonal antisera (8, 37).

To assess proteinase K sensitivity of individual proteins, 15 µg of purified GerAA^NTD^, GerBC, and GerD were treated with proteinase K (14 µg/mL) at room temperature for various time points. Reactions were quenched with PMSF, and one quarter of each was analyzed by SDS-PAGE and Coomassie staining.

**Protein modeling**

Structural models of GR proteins were predicted using AlphaFold 3 (38). Structural alignments and rendering of molecules were conducted with PyMOL (Schrödinger, Inc.). Structural coordinates for inosine were obtained from the Cambridge Structural Database and aligned with hydantoin using PyMOL’s Pair Fit tool. Chai-1 (39) was also used to predict potential inosine, L*-*alanine, and Na^+^ binding sites in GerIB and GerQB.

**Table S1.** Summary of crystallographic analysis.

| GerIA^NTD^ | SeMet-MAD (Peak) | SeMet-MAD (Inflection) | |  |
| --- | --- | --- | --- | --- |
| **Data collection** |  |  | |  |
| Wavelength (Å) | 0.97929 | 0.97950 | |  |
| Space group | *C 2 2 21* | *C 2 2 21* | |  |
| Cell dimensions (Å) |  |  | |  |
| *a*, *b*, *c* (Å) | 82.17, 122.88, 212.20 | 82.32, 123.00, 212.33 | |  |
| *α*, β, γ (°) | 90.0, 90.0, 90.0 | 90.0, 90.0, 90.0 | |  |
| Resolution (Å) | 38.31-2.80 (2.95-2.80) | 38.38-2.94 (3.12-2.94) | |  |
| *R*_sym_ (%) | 0.28 (2.90) | 0.35 (3.54) | |  |
| Mean (*I*/σ*I*) | 16.0 (2.4) | 15.9 (2.4) | |  |
| CC_1/2_ | 99.9 (93.4) | 99.9 (93.4) | |  |
| Completeness (%) | 100 (100) | 100 (100) | |  |
| Multiplicity | 39.6 (38.8) | 39.5 (40.1) | |  |
| **Refinement** | |  | | |
| Resolution (Å) | 2.80-38.30 |  |  |  |
| No. reflections (\|F\|>0σ) | 26,875 |  |  |  |
| *R*_work_/*R*_free_ (%) | 19.9/22.9 |  |  |  |
| No. atoms |  |  |  |  |
| Protein | 5,798 |  |  |  |
| Water | 162 |  |  |  |
| Average B-factors (Å^2^) |  |  |  |  |
| Protein | 88.22 |  |  |  |
| Water | 73.06 |  |  |  |
| Wilson B-factors (Å^2^) | 74.03 |  |  |  |
| R.m.s. deviations |  |  |  |  |
| Bond lengths (Å) | 0.01 |  |  |  |
| Bond angles (°) | 1.17 |  |  |  |
| Ramachandran plot (%) |  |  |  |  |
| Favored (%) | 96.0 |  |  |  |
| Allowed (%) | 4.0 |  |  |  |
| Outliers (%) | 0.0 |  |  |  |

Values in parentheses are for the highest-resolution shell. MAD, multiple wavelength anomalous dispersion.

| **CSP Ranking** | **Residues** | **Location** |
| --- | --- | --- |
| 1 | Tyr266 | N1/S1 |
| 2 | Ile278 | N1/S2 |
| 3 | Thr465 | N2/H10 |
| 4 | Val377 | Linker |
| 5 | Asn265 | N1/S1 |
| 6 | Glu463 | N2/S8 |
| 7 | Tyr280 | N1/S2 |
| 8 | Ile327 | N1/S2 |
| 9 | Asn356 | Linker |
| 10 | Phe406 | N2/S6-S7 |
| 11 | Val264 | N1/S1 |
| 12 | Arg358 | Linker |
| 13 | Ser279 | N1/S2 |
| 14 | Gly404 | N2/S6 |
| 15 | Phe239 | N1 |
| 16 | Ile338 | N1/S4 |
| 17 | Lys453 | N2/H9-S8 |
| 18 | Thr240 | N1 |
| 19 | Val468 | N1/H10 |
| 20 | Tyr357 | Linker |

**Table S2.** Ranking of GerIA^NTD^ residues by magnitude of CSP upon inosine titration.

Residues in navy were subjected to SDM analyses.

**Table S3.** *Bacillus cereus* strains used in this work.

| Strain | Genotype | Source |
| --- | --- | --- |
| ATCC 10876 | Wild type | Anne Moir |
| GOV15 | *gerI* | This work |
| GOV16 | *gerQ* | This work |
| GOV17 | *gerI gerQ* | This work |
| GerI variant strains^b^ | |  |
| GOV18 | pHT-P*_gerI_*-*gerIA*-*gfp-gerIB-gerIC- gerID* | This work |
| GOV19 | pHT-P*_gerI_*-*gerIA^Y266A^*- *gfp-gerIB-gerIC-gerID* | This work |
| GOV20 | pHT-P*_gerI_*-*gerIA^I278A^*- *gfp-gerIB-gerIC-gerID* | This work |
| GOV21 | pHT-P*_gerI_*-*gerIA^Y280A^*- *gfp-gerIB-gerIC-gerID* | This work |
| GOV22 | pHT-P*_gerI_*-*gerIA^I388A^*- *gfp-gerIB-gerIC-gerID* | This work |
| GOV23 | pHT-P*_gerI_*-*gerIA^I388V^*- *gfp-gerIB-gerIC-gerID* | This work |
| GOV24^c^ | pHT-P*_gerI_*-*gerIA^D355-P363^*- *gfp-gerIB-gerIC-gerID* | This work |
| GOV25 | pHT-P*_gerI_*-*gerIA^Y266A/Y280A^*- *gfp-gerIB-gerIC-gerID* | This work |
| GOV26 | pHT-P*_gerI_*-*gerIA*- *gfp-gerIB^Q23A^-gerIC-gerID* | This work |
| GOV27 | pHT-P*_gerI_*-*gerIA*- *gfp-gerIB^F24A^-gerIC-gerID* | This work |
| GOV28 | pHT-P*_gerI_*-*gerIA*- *gfp-gerIB^G25A^-gerIC-gerID* | This work |
| GOV29 | pHT-P*_gerI_*-*gerIA*- *gfp-gerIB^F31A^-gerIC-gerID* | This work |
| GOV30 | pHT-P*_gerI_*-*gerIA*- *gfp-gerIB^Y95A^-gerIC-gerID* | This work |
| GOV31 | pHT-P*_gerI_*-*gerIA*- *gfp-gerIB^V99A^-gerIC-gerID* | This work |
| GOV32 | pHT-P*_gerI_*-*gerIA*- *gfp-gerIB^Y193A^-gerIC-gerID* | This work |
| GOV33 | pHT-P*_gerI_*-*gerIA*- *gfp-gerIB^M195A^-gerIC-gerID* | This work |
| GOV34 | pHT-P*_gerI_*-*gerIA*- *gfp-gerIB^T196A^-gerIC-gerID* | This work |
| GOV35 | pHT-P*_gerI_*-*gerIA*- *gfp-gerIB^Y229A^-gerIC-gerID* | This work |
| GOV36 | pHT-P*_gerI_*-*gerIA*- *gfp-gerIB^Y275A^-gerIC-gerID* | This work |
| GOV37 | pHT-P*_gerI_*-*gerIA*- *gfp-gerIB^S282A^-gerIC-gerID* | This work |
| GOV38 | pHT-P*_gerQ_*-*gerQA*-*gfp-gerQB-gerQC- gerQD* | This work |

^a^The *gerI* and *gerQ* gene sequences refer to the *B. cereus* ATCC 14579 operons.

^b^These strains are resistant to erythromycin (1 µg/mL) and lincomycin (25 µg/mL).

^c^Amino acid deletions from D355 to P363 (linker deletion).

**Table S4.** *B. cereus* ATCC 14579 GerI site-directed mutagenesis summary.

| **A subunit proteins** | | | | | | |
| --- | --- | --- | --- | --- | --- | --- |
| GerIA substitution | GerQ equivalent | Location | NMR chemical shift perturbation rank | Germinosome  (GerIA-GFP) | Germination in *gerI gerQ* background | Germination in *gerI* background |
| Uniprot: Q816T6 | Uniprot: Q81BQ2 |  |  |  |  |  |
| Y266A | L31 | NTD | CSP 1 | **+** | Approx. 55% DPA release. | Restored to WT levels |
| I278A | I42 | NTD | CSP 2 | **-** | <5% DPA release | Approx. 15% of WT levels |
| Y280A | Y44 | NTD | CSP 7 | **+** | Approx. 75% DPA release | Approx. 90% WT levels |
| I338A | I102 | NTD | CSP 16 | **-** | <5% DPA release | Approx. 10% WT levels |
| I338V | I102 | NTD | CSP 16 | **+** | DPA release similar to WT | Didn’t test |
| Y266A/ Y280A |  | NTD | CSP 1 and 7 | **-** | <5% DPA release | Didn’t test |
| Linker  D355 – P363 deletion |  | NTD Interdomain loop | N356, Y357, R358 NMR CSP 9, 20 & 12. | **+** | <5% DPA release | <5% DPA release |

| **B subunit proteins** | | | | | | |
| --- | --- | --- | --- | --- | --- | --- |
| GerIB  Uniprot: Q816T5 | GerQB  Uniprot: Q81BQ3 | Location | Comment | Germinosome  (GerIA-GFP) | Germination in *gerI gerQ* background | Germination in *gerI* background |
| Q23A | Q23 | TM1 | Residue precedes unwound region. Pointing towards inosine. | + | DPA release similar to WT | Restored to WT levels |
| F24A | M24 | TM1 | Unwound region. Carbonyl on backbone could interact with ion. | + | <10% DPA. | Very slow incline to <10% WT |
| G25A | G25 | TM1 | Unwound region. No obvious interaction with ion from carbonyl. | + | <5% DPA release. | Flat DPA |
| F31A | F31 | TM1 | Interact with hypoxanthine moiety of inosine? | + | <25% DPA. | Restored to WT levels |
| Y95A | Y95 | TM3 | Interact with ribose moiety of inosine? | + | Approx. 60% DPA. | Restored to WT levels |
| V99A | L99A | TM3 | Very close to inosine ribose, less so to alanine. | + | DPA release more rapid than WT. Essential residue in *B. subtilis* GerAB | WT levels |
| Y193A | F193 | TM6 | Close to unwound region. Steric clash with inosine in overlay model. | + | <5% DPA. | Slowly gets up to 33% WT level |
| M195A | F195 | TM6 | Unwound region. Interact with hypoxanthine? | + | DPA release slightly faster than WT. | WT levels |
| T196A | F196 | TM6 | Unwound region. Interact with inosine and alanine. | + | DPA release more rapid than WT. Important in *B. subtilis* GerAB. | WT levels |
| Y229A | Y229 | TM7 | Interact with alanine? | + | Flat DPA. | Restored to WT levels |
| Y275A | W275 | TM8 | Interact with inosine? | + | Slow; 25 % DPA | Restored to WT levels |
| S282A | N282 | TM8 | Close to bound cation. | + | DPA release similar to WT. Important in *B. subtilis* GerAB. | WT levels |

**
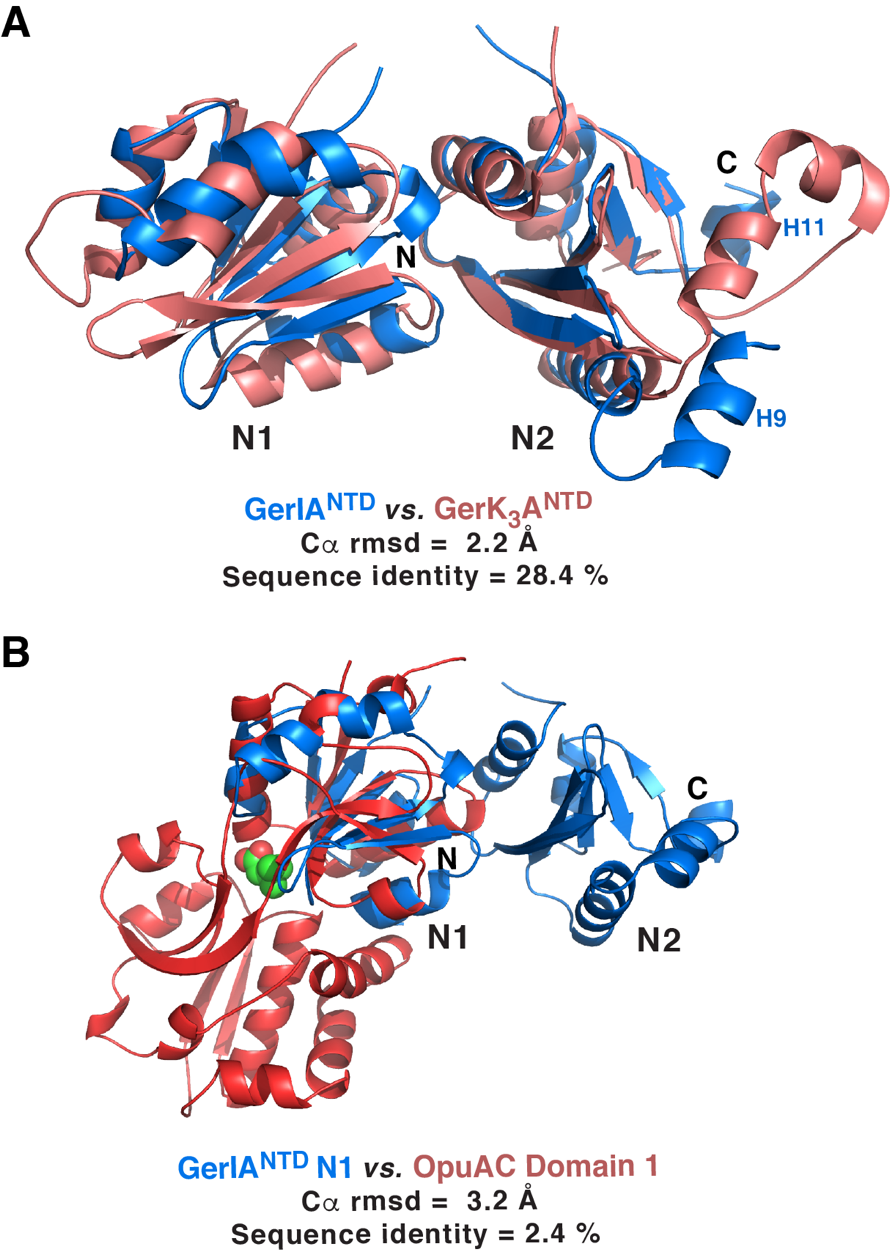
Fig. S1.** Structural comparison of GerIA^NTD^, GerK_3_A^NTD^ and OpuAC. (A) Superimposition of GerIA^NTD^ (blue) and GerK_3_A^NTD^ (red; PDB ID: 6O59). (B) Superimposition of the N1 domain of GerIA^NTD^ (blue) and the domain 1 of OpuAC (red; PDB ID: 2B4L). The bound ligand for OpuAC, glycine betaine, is shown in space-filling representation.

**
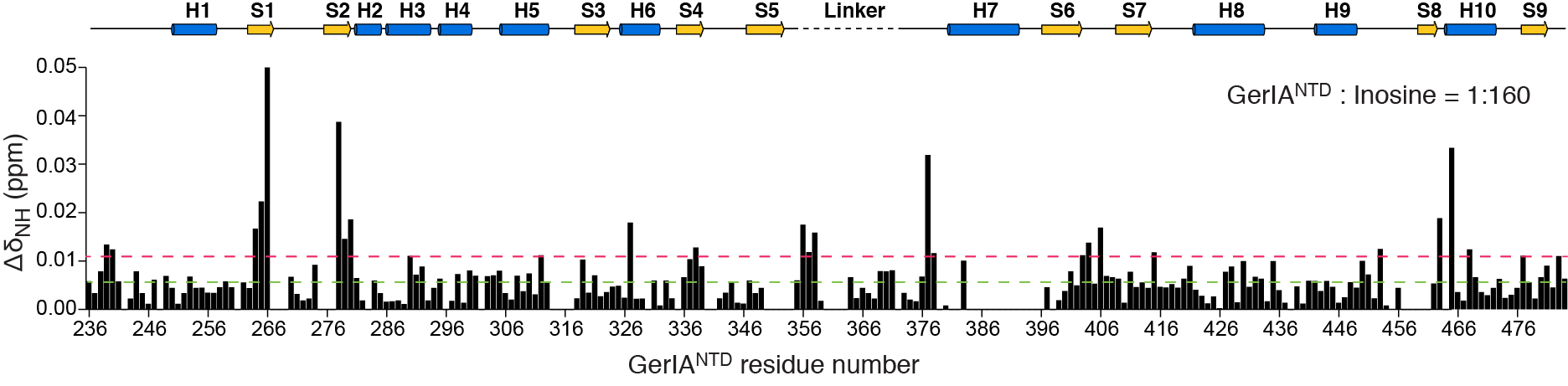
**

**Fig. S2.** Per-residue backbone NH chemical shift differences of free and inosine-titrated GerIA^NTD^. Dashed green and red lines indicate the threshold of one (1σ) and two SDs (2σ, 0.005 ppm) calculated across all assigned residues, respectively. Secondary-structure assignments from the crystal structure are shown as blue cylinders (α helices) and orange arrows (β strands), while disordered regions are shown as dashed lines.


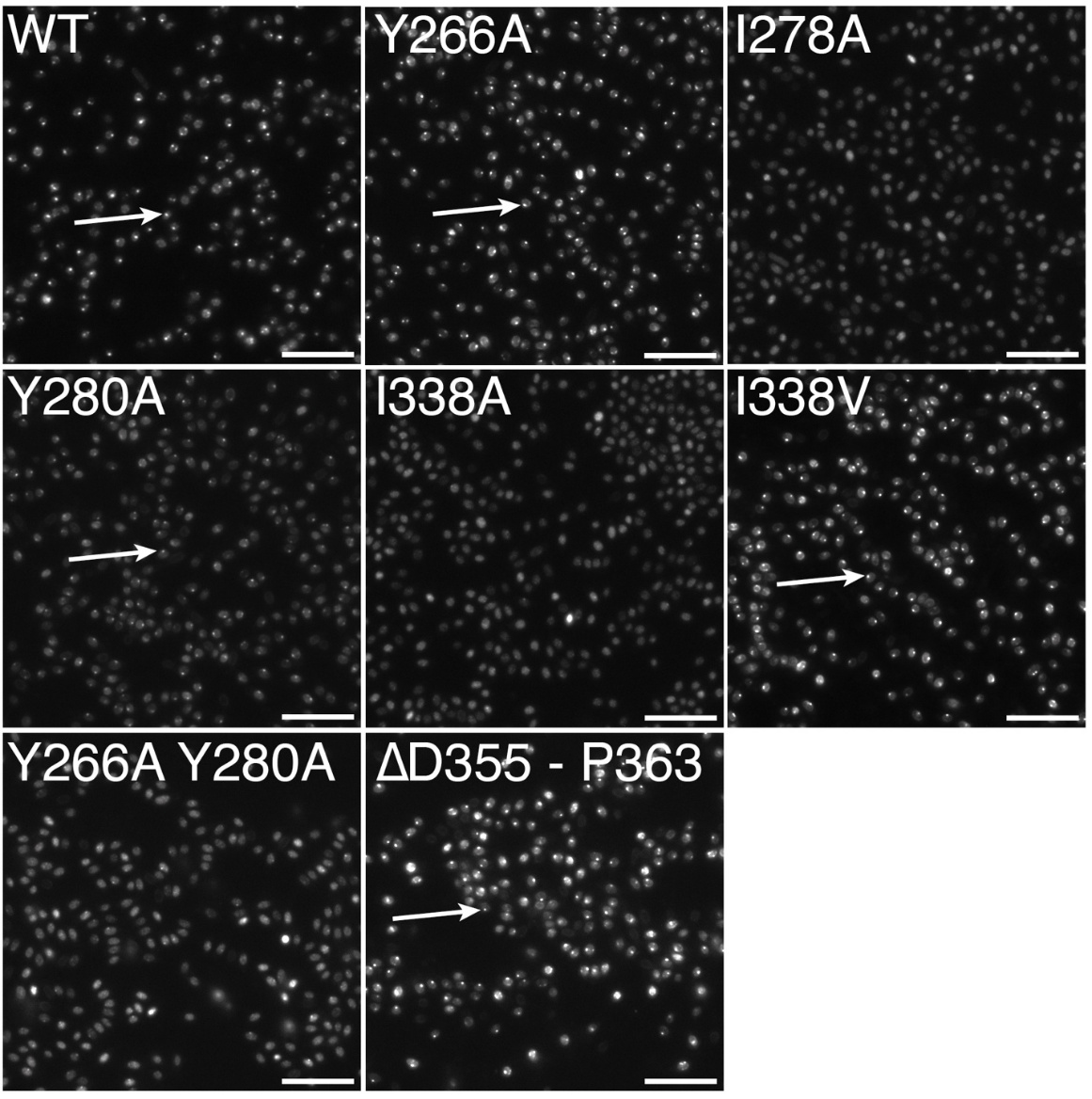


**Fig. S3.** Germinosome analysis of *B. cereus* *gerI gerQ* spores with GerIA-GFP variants. Freshly prepared and purified spores were placed on cover slips and imaged with an Olympus BX53 microscope fitted with a 100× 1.30 NA oil objective lens and filters for GFP. GerIA substitutions are labelled in the top left of each panel. Arrows point to germinosomes in individual spores.

**
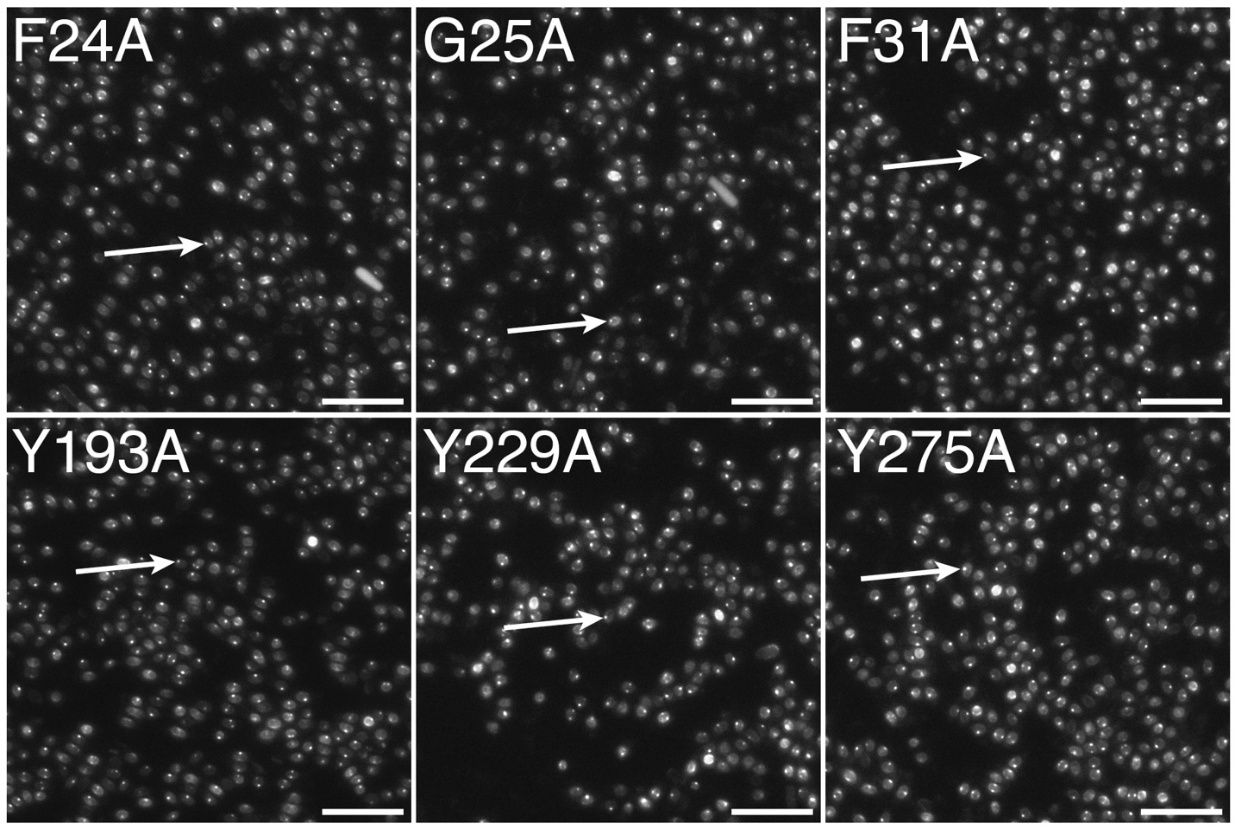
**

**Fig. S4.** Germinosome analysis of *B. cereus* *gerI gerQ* spores with GerIB variants. Freshly prepared and purified spores (2 µl) were placed on cover slips and imaged with an Olympus BX53 microscope fitted with a 100× 1.30 NA oil objective lens and filters for GFP. GerIB substitutions are labelled in the top left of each panel. The fluorescence signal is from GerIA-GFP. Arrows point to germinosomes in individual spores.

**
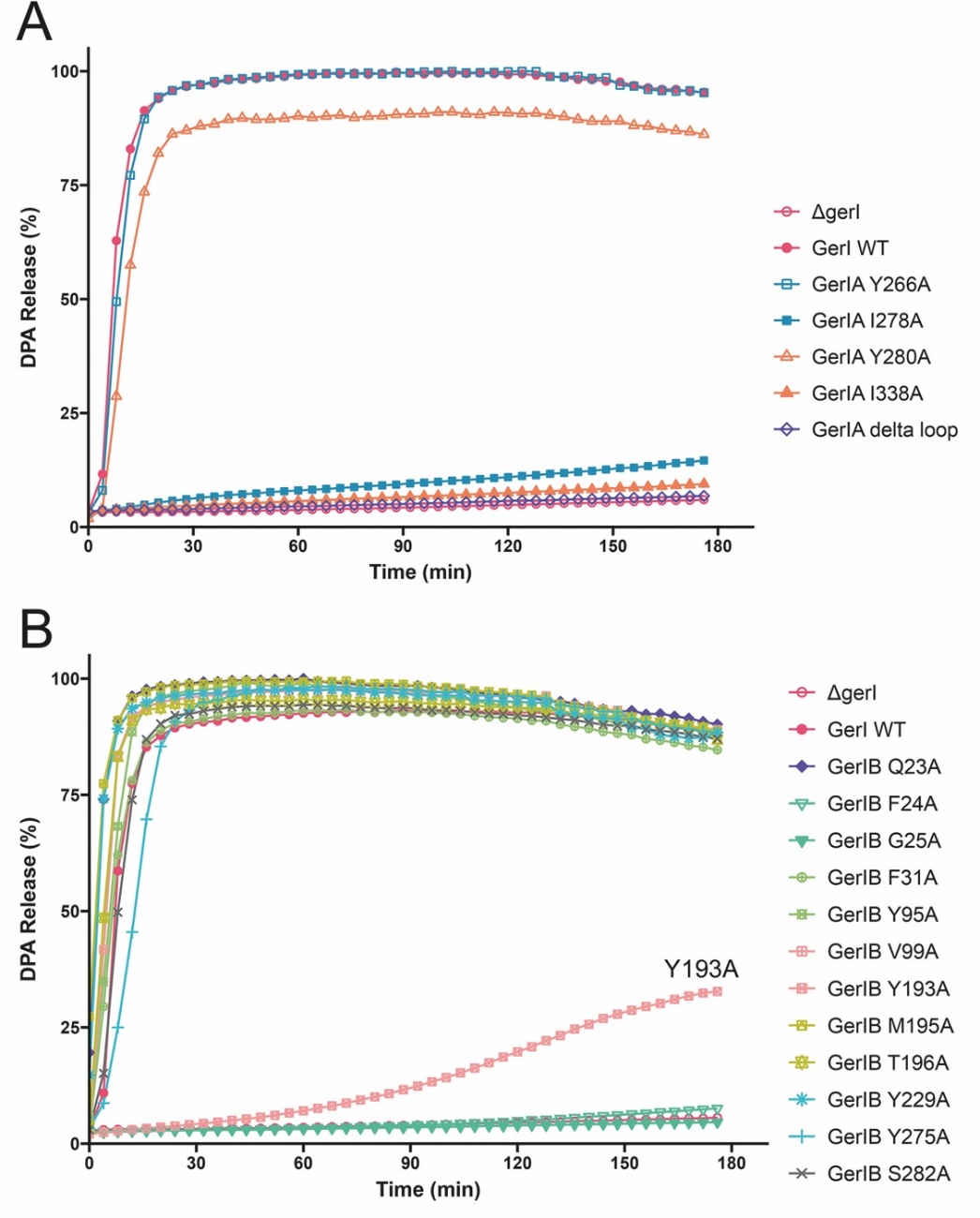
**

**Fig. S5.** Germination of *B. cereus gerI* spores with ectopic plasmid encoded (A) GerIA and (B) GerIB variants. Spores were heat-shocked (75°C for 30 min) and cooled before resuspending in buffer (50 mM Tris-HCl, 100 mM NaCl, pH 8.0, supplemented with 1 mM inosine and 50 µM TbCl_3_). Germination was monitored using fluorimetric measurements of DPA release as described in SM Materials and Methods. The presented data are average values from triplicate experiments conducted with the same batch of spores. For clarity, GerIA I338A and Δloop (D355-P363), and GerIB F24A and G25A measurements are indistinguishable from the baseline Δ*gerI* spores.

**
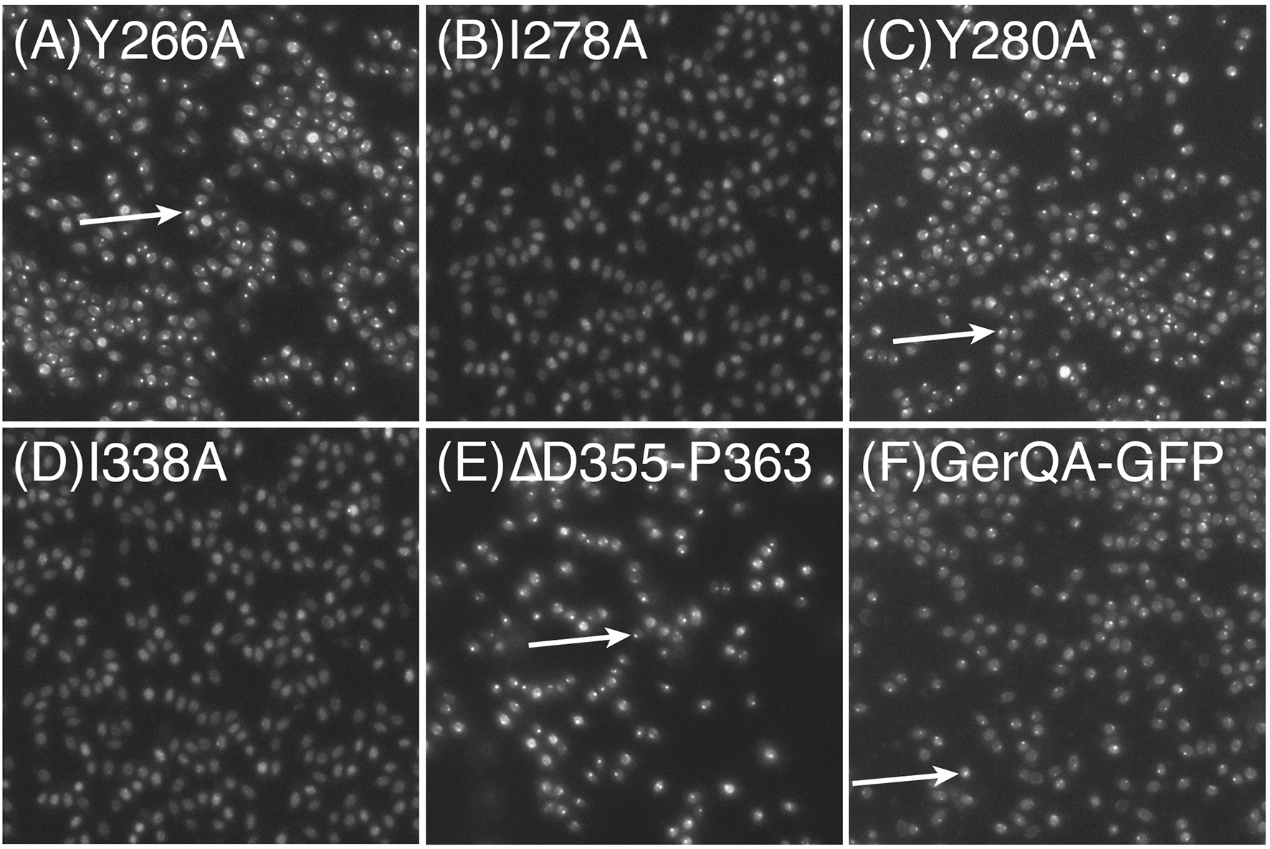
**

**Fig. S6.** Germinosome analysis of *B. cereus* *gerI* spores with (A – E) variant GerIA-GFP, and (F) GerQA-GFP proteins. Freshly prepared and purified spores were placed on cover slips and imaged with an Olympus BX53 microscope fitted with a 100× 1.30 NA oil objective lens and filters for GFP. GerIA and GerQA substitutions are labelled in the top left of each panel. Arrows point to germinosomes in individual spores.


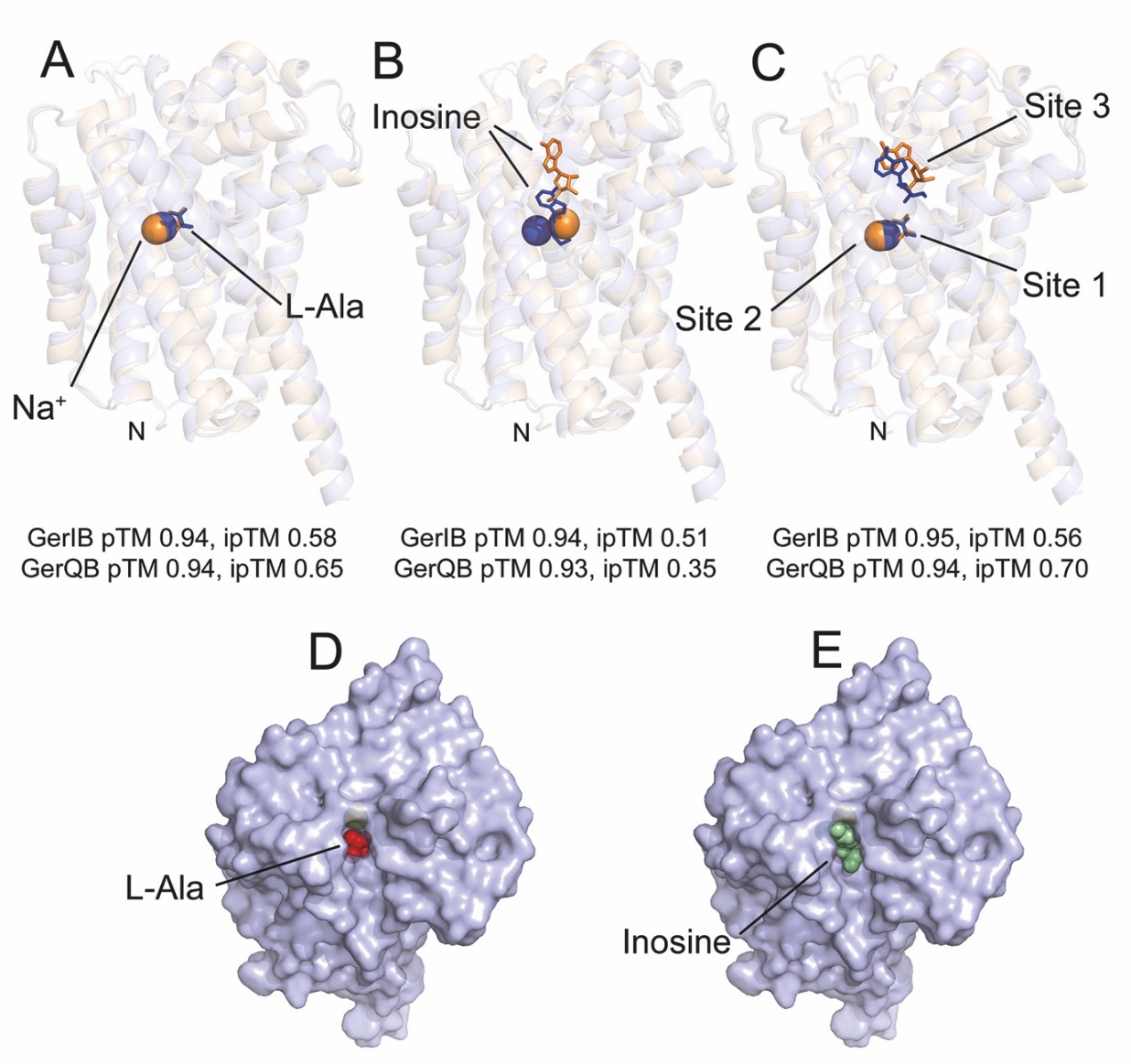


**Fig. S7.** Predicted germinant binding sites in *B. cereus* ATCC 14579 GerIB and GerQB. Superimposed Chai-1 structural predictions of GerIB (blue) with GerQB (orange) with bound (A) Na^+^ and L-alanine, (B) Na^+^ and inosine, and (C) Na^+^, L-alanine and inosine (labelled with recurring binding Sites 1, 2 and 3). Site 1 is most frequently predicted by the Chai-1 algorithm to represent an amino acid binding site, Site 2 is the cation binding site, and Site 3 accommodates inosine. Presented pTM values are commensurate with confidently predicted overall structures. Confidence in predicted ligand binding sites (ipTM) is highest when inosine, L-alanine and Na^+^ are all present in GerQB (panel C) and lowest when L-alanine is absent (panel B). (D) Top-down surface representation view of GerIB showing the entrance to the bound L-alanine molecule (red), and then (E) with inosine additionally bound.

**
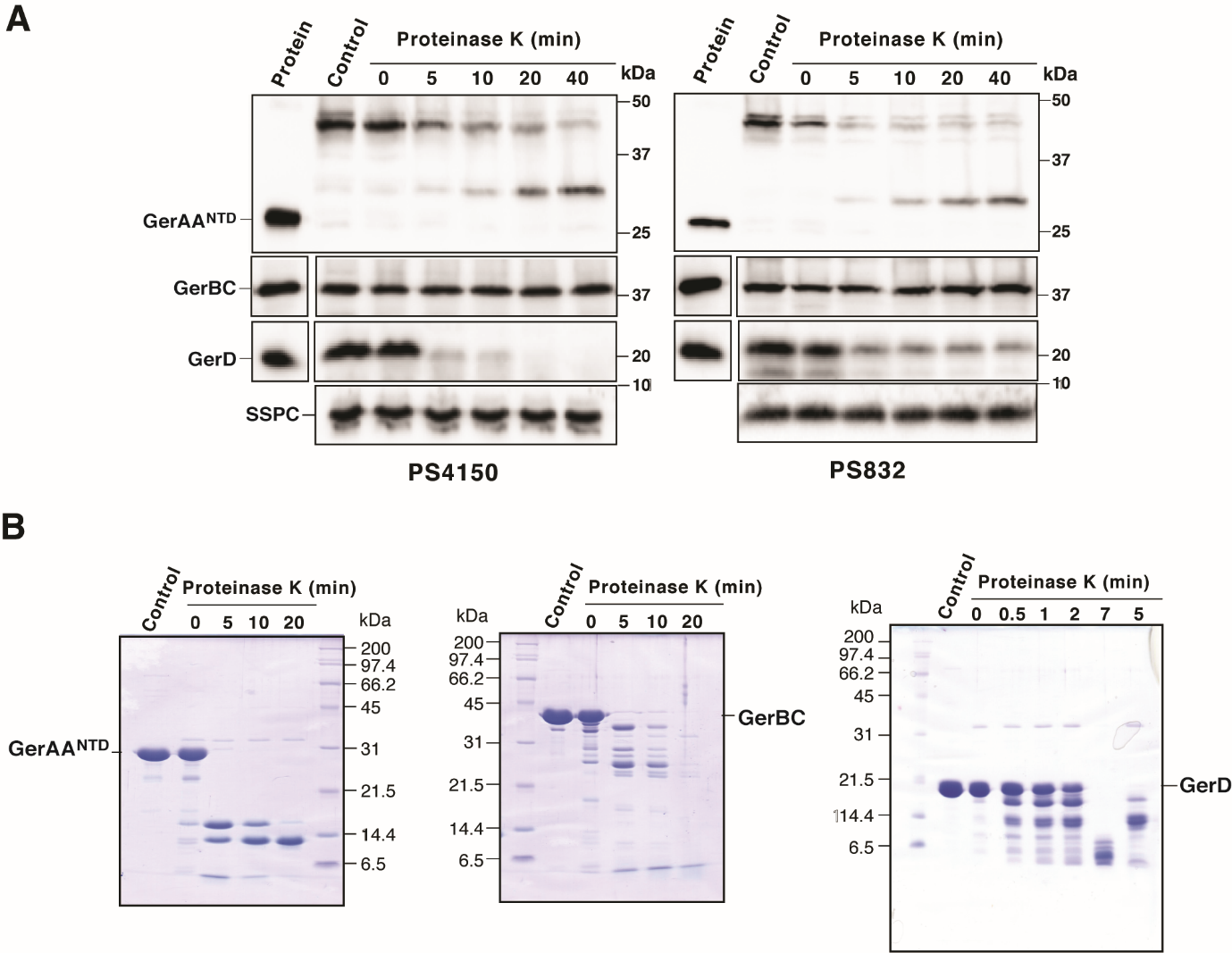
**

**Fig. S8.** Effects of proteinase K treatment on *B. subtilis* GerAA, GerBC and GerD proteins. (A) Heat-activated PS4150 (left) and decoated PS832 (right) spores were germinated as described in SM Materials and Methods. Equal aliquots of pelleted germinated spores were incubated without (control) or with proteinase K for 0, 5, 10, 20, or 40 mins, after which the proteinase K was neutralized. The samples, along with aliquots of purified GerAA^NTD^, GerBC and GerD proteins, were assayed by Western blotting using their respective antibodies. Levels of spore core protein SSPC were assayed as loading control.

(B) *B. subtilis* GerAA^NTD^, GerBC, and GerD proteins were expressed and purified as described in SM Materials and Methods. Equal aliquots of purified proteins were incubated without (control) or with proteinase K for various time points, after which the proteinase K was neutralized. The samples were assayed by SDS-PAGE and Coomassie Blue staining.


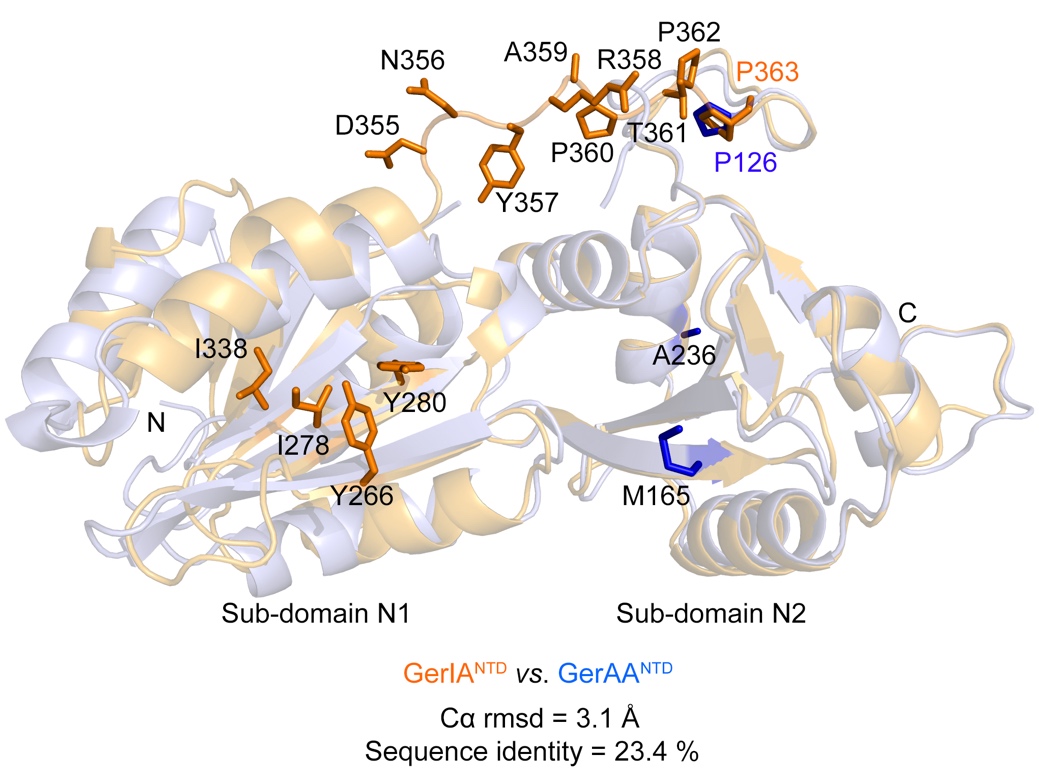


**Fig. S9.** *FATCAT* (40) flexible pairwise structural alignment between *B. cereus* ATCC 14579 GerIA^NTD^ (orange) and *B. subtilis* GerAA^NTD^ (blue). The alignment has an optimized RMSD of 3.1 Å when a single twist is introduced in the AlphaFold predicted GerAA^NTD^ structure. Highlighted residues with sidechains shown as sticks are of functional or structural importance as demonstrated for GerIA^NTD^ in the present study or for GerAA in (41).

**
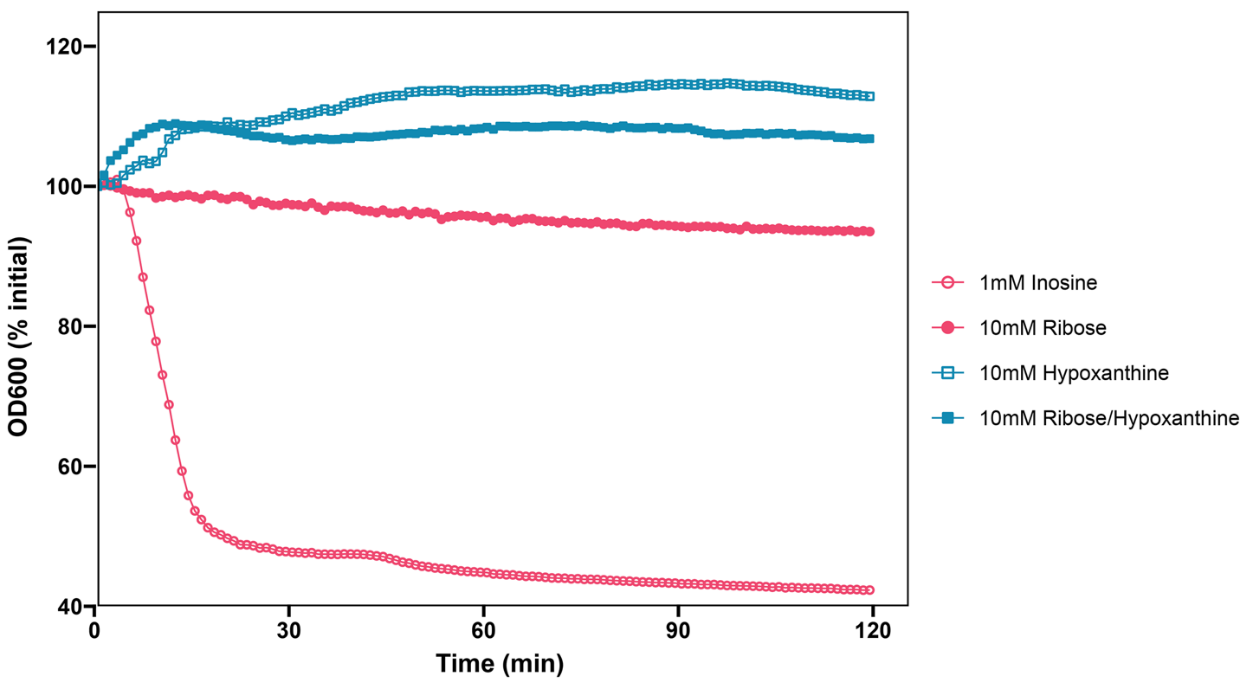
**

**Fig. S10.** Germination of *B. cereus* 10876 spores in 50 mM Tris-HCl, 100 mM NaCl, pH 8.0, supplemented with (i) 1 mM inosine, (ii) 10 mM ribose, (iii) 10 mM hypoxanthine, or (iv) 10 mM of both ribose and hypoxanthine. Spores were heat-shocked (75°C for 30 min) and cooled before resuspending in buffer, and germination was monitored by measuring absorbance at OD_600_. The presented data are average values from triplicate experiments conducted with the same batch of spores. Similar values were obtained with additional batches of spores. SD from mean values is <10%.

**SM REFERENCES**

1. Gao Y, Amon JD, Artzi L, Ramirez-Guadiana FH, Brock KP, Cofsky JC, Marks DS, Kruse AC, Rudner DZ. 2023. Bacterial spore germination receptors are nutrient-gated ion channels. *Science* 380(6643):387-391.

2. Wilson MJ, Carlson PE, Janes BK, Hanna PC. 2012. Membrane topology of the *Bacillus anthracis* GerH germinant receptor proteins. *J Bacteriol* 194(6):1369-77.

3. Korza G, Setlow P. 2013. Topology and accessibility of germination proteins in the *Bacillus subtilis* spore inner membrane. *J Bacteriol* 195(7):1484-91.

4. Li Y, Setlow B, Setlow P, Hao B. 2010. Crystal structure of the GerBC component of a *Bacillus subtilis* spore germinant receptor. *J Mol Biol* 402(1):8-16.

5. Igarashi T, Setlow B, Paidhungat M, Setlow P. 2004. Effects of a gerF (lgt) mutation on the germination of spores of *Bacillus subtilis*. *J Bacteriol* 186(10):2984-91.

6. Griffiths KK, Zhang J, Cowan AE, Yu J, Setlow P. 2011. Germination proteins in the inner membrane of dormant *Bacillus subtilis* spores colocalize in a discrete cluster. *Mol Microbiol* 81(4):1061-77.

7. Wang Y, Breedijk RMP, Hink MA, Bults L, Vischer NOE, Setlow P, Brul S. 2021. Dynamics of germinosome formation and fret-based analysis of interactions between GerD and germinant receptor subunits in *Bacillus cereus* spores. *Int J Mol Sci* 22(20).

8. Li Y, Jin K, Ghosh S, Devarakonda P, Carlson K, Davis A, Stewart KA, Cammett E, Pelczar Rossi P, Setlow B, Lu M, Setlow P, Hao B. 2014. Structural and functional analysis of the GerD spore germination protein of *Bacillus* species. *J Mol Biol* 426(9):1995-2008.

9. Flores MJ, Duricy K, Choudhary S, Laue M, Popham DL. 2023. A family of spore lipoproteins stabilizes the germination apparatus by altering inner spore membrane fluidity in *Bacillus subtilis* spores. *J Bacteriol* 205(10):e0014223.

10. Ramirez-Peralta A, Gupta S, Butzin XY, Setlow B, Korza G, Leyva-Vazquez MA, Christie G, Setlow P. 2013. Identification of new proteins that modulate the germination of spores of *Bacillus* species. *J Bacteriol* 195(13):3009-21.

11. Van Duyne GD, Standaert RF, Karplus PA, Schreiber SL, Clardy J. 1993. Atomic structures of the human immunophilin FKBP-12 complexes with FK506 and rapamycin. *J Mol Biol* 229(1):105-24.

12. Kabsch W. 2010. Xds. *Acta Crystallogr D Biol Crystallogr* 66(Pt 2):125-32.

13. Winter G, McAuley KE. 2011. Automated data collection for macromolecular crystallography. *Methods* 55(1):81-93.

14. Grosse-Kunstleve RW, Adams PD. 2003. Substructure search procedures for macromolecular structures. *Acta Crystallogr D Biol Crystallogr* 59(Pt 11):1966-73.

15. Terwilliger TC, Berendzen J. 1999. Automated MAD and MIR structure solution. *Acta Crystallogr D Biol Crystallogr* 55(Pt 4):849-61.

16. McCoy AJ, Grosse-Kunstleve RW, Adams PD, Winn MD, Storoni LC, Read RJ. 2007. Phaser crystallographic software. *J Appl Crystallogr* 40(Pt 4):658-674.

17. Emsley P, Lohkamp B, Scott WG, Cowtan K. 2010. Features and development of Coot. *Acta Crystallogr D Biol Crystallogr* 66(Pt 4):486-501.

18. Murshudov GN, Vagin AA, Dodson EJ. 1997. Refinement of macromolecular structures by the maximum-likelihood method. *Acta Crystallogr D Biol Crystallogr* 53(Pt 3):240-55.

19. Winn MD, Ballard CC, Cowtan KD, Dodson EJ, Emsley P, Evans PR, Keegan RM, Krissinel EB, Leslie AG, McCoy A, McNicholas SJ, Murshudov GN, Pannu NS, Potterton EA, Powell HR, Read RJ, Vagin A, Wilson KS. 2011. Overview of the CCP4 suite and current developments. *Acta Crystallogr D Biol Crystallogr* 67(Pt 4):235-42.

20. Williams CJ, Headd JJ, Moriarty NW, Prisant MG, Videau LL, Deis LN, Verma V, Keedy DA, Hintze BJ, Chen VB, Jain S, Lewis SM, Arendall WB, 3rd, Snoeyink J, Adams PD, Lovell SC, Richardson JS, Richardson DC. 2018. MolProbity: More and better reference data for improved all-atom structure validation. *Protein Sci* 27(1):293-315.

21. Li Y, Jin K, Perez-Valdespino A, Federkiewicz K, Davis A, Maciejewski MW, Setlow P, Hao B. 2019. Structural and functional analyses of the N-terminal domain of the A subunit of a *Bacillus megaterium* spore germinant receptor. *Proc Natl Acad Sci USA* 116(23):11470-11479.

22. Delaglio F, Grzesiek S, Vuister GW, Zhu G, Pfeifer J, Bax A. 1995. NMRPipe: a multidimensional spectral processing system based on UNIX pipes. *J Biomol NMR* 6(3):277-93.

23. Vranken WF, Boucher W, Stevens TJ, Fogh RH, Pajon A, Llinas M, Ulrich EL, Markley JL, Ionides J, Laue ED. 2005. The CCPN data model for NMR spectroscopy: development of a software pipeline. *Proteins* 59(4):687-96.

24. Maciejewski MW, Schuyler AD, Gryk MR, Moraru, II, Romero PR, Ulrich EL, Eghbalnia HR, Livny M, Delaglio F, Hoch JC. 2017. NMRbox: A Resource for Biomolecular NMR Computation. *Biophys J* 112(8):1529-1534.

25. Schumann FH, Riepl H, Maurer T, Gronwald W, Neidig KP, Kalbitzer HR. 2007. Combined chemical shift changes and amino acid specific chemical shift mapping of protein-protein interactions. *J Biomol NMR* 39(4):275-89.

26. Mulder FA, Schipper D, Bott R, Boelens R. 1999. Altered flexibility in the substrate-binding site of related native and engineered high-alkaline *Bacillus* subtilisins. *J Mol Biol* 292(1):111-23.

27. Ulrich EL, Akutsu H, Doreleijers JF, Harano Y, Ioannidis YE, Lin J, Livny M, Mading S, Maziuk D, Miller Z, Nakatani E, Schulte CF, Tolmie DE, Kent Wenger R, Yao H, Markley JL. 2008. BioMagResBank. *Nucleic Acids Res* 36(Database issue):D402-8.

28. Ghosh A, Manton JD, Mustafa AR, Gupta M, Ayuso-Garcia A, Rees EJ, Christie G. 2018. Proteins encoded by the *gerP* operon are localized to the inner coat in *Bacillus* *cereus* spores and are dependent on GerPA and SafA for assembly. *Appl Environ Microbiol* 84(14):aem.00760-18.

29. Janes BK, Stibitz S. 2006. Routine markerless gene replacement in *Bacillus anthracis*. *Infect Immun* 74(3):1949-53.

30. Arantes O, Lereclus D. 1991. Construction of cloning vectors for *Bacillus thuringiensis*. *Gene* 108(1):115-9.

31. Hindle AA, Hall EA. 1999. Dipicolinic acid (DPA) assay revisited and appraised for spore detection. *Analyst* 124(11):1599-604.

32. Ghosh S, Setlow B, Wahome PG, Cowan AE, Plomp M, Malkin AJ, Setlow P. 2008. Characterization of spores of *Bacillus subtilis* that lack most coat layers. *J Bacteriol* 190(20):6741-8.

33. Paidhungat M, Setlow B, Driks A, Setlow P. 2000. Characterization of spores of *Bacillus subtilis* which lack dipicolinic acid. *J Bacteriol* 182(19):5505-12.

34. Nicholson WL, Setlow P. 1990. Sporulation, germination and outgrowth, p 391-450. *In* Harwood CR, Cutting SM (ed), Molecular Biological Methods for *Bacillus*. John Wiley and Sons, Chichester, U.K.

35. Schuck P. 2000. Size-distribution analysis of macromolecules by sedimentation velocity ultracentrifugation and lamm equation modeling. *Biophys J* 78(3):1606-19.

36. Laue TM, Shah BD, Ridgeway TM, Pelletier SL. 1992. Computer-aided interpretation of analytical sedimentation data for proteins., p 90-125. *In* Harding S, Rowe A, Horton J (ed), Analytical Ultracentrifugation in Biochemistry and Polymer Science. Royal Society of Chemistry Press, Cambridge, U.K.

37. Setlow B, Setlow P. 1979. Localization of low-molecular-weight basic proteins in *Bacillus megaterium* spores by cross-linking with ultraviolet light. *J Bacteriol* 139(2):486-94.

38. Abramson J, Adler J, Dunger J, Evans R, Green T, Pritzel A, Ronneberger O, Willmore L, Ballard AJ, Bambrick J, Bodenstein SW, Evans DA, Hung C-C, O’Neill M, Reiman D, Tunyasuvunakool K, Wu Z, Žemgulytė A, Arvaniti E, Beattie C, Bertolli O, Bridgland A, Cherepanov A, Congreve M, Cowen-Rivers AI, Cowie A, Figurnov M, Fuchs FB, Gladman H, Jain R, Khan YA, Low CMR, Perlin K, Potapenko A, Savy P, Singh S, Stecula A, Thillaisundaram A, Tong C, Yakneen S, Zhong ED, Zielinski M, Žídek A, Bapst V, Kohli P, Jaderberg M, Hassabis D, Jumper JM. 2024. Accurate structure prediction of biomolecular interactions with AlphaFold 3. *Nature* 630(8016):493-500.

39. Boitreaud J, Dent J, McPartlon M, Meier J, Reis V, Rogozhnikov A, Wu K. 2024. Chai-1: Decoding the molecular interactions of life. *bioRxiv* doi:10.1101/2024.10.10.615955:2024.10.10.615955.

40. Ye Y, Godzik A. 2004. FATCAT: a web server for flexible structure comparison and structure similarity searching. *Nucleic Acids Res* 32(Web Server issue):W582-5.

41. Amon JD, Artzi L, Rudner DZ. 2022. Genetic evidence for signal transduction within the *Bacillus subtilis* GerA germinant receptor. *J Bacteriol* 204(2):e0047021.
